# The Association Between Educational Attainment and Non-Alcoholic Fatty Liver Disease: A Systematic Review and Meta-Analysis of Observational Studies

**DOI:** 10.3390/healthcare14091197

**Published:** 2026-04-29

**Authors:** Yaolong Xu, Jiaxin Zhao, Ligang Yang

**Affiliations:** Key Laboratory of Environmental Medicine and Engineering of Ministry of Education, Department of Nutrition and Food Hygiene, School of Public Health, Southeast University, Nanjing 210003, China

**Keywords:** educational attainment, non-alcoholic fatty liver disease (NAFLD), social determinant, meta-analysis, socioeconomic status

## Abstract

**Highlights:**

**What are the main findings?**

- Higher educational attainment would be positively associated with
- NAFLD risk in some cases, and the underlying mechanisms warrant
- further investigation.

**What are the implications of the main findings?**

- Clinical and public health practice should not neglect individuals with
- higher educational levels when implementing targeted liver screening
- preventive strategies for NAFLD.

**Abstract:**

**Objectives:** Educational attainment appears to be associated with non-alcoholic fatty liver disease (NAFLD). The inconsistent findings across existing studies necessitate a thorough meta-analysis to elucidate this association. **Methods:** A systematic search of PubMed, Web of Science, and Scopus was conducted from inception to 31 December 2024, without language restrictions. Data were analyzed using Review Manager 5.4, with pooled odds ratios (ORs) and 95% confidence intervals (CIs) estimated via appropriate models. **Results:** 27 studies involving 446,312 participants (93,116 NAFLD; 353,196 healthy individuals) were included. Noteworthy heterogeneity was detected, with I^2^ = 96% for more-than-high-school and I^2^ = 95% for high-school-education when we pooled all the studies together. Further subgroup analyses suggested that higher education was inversely associated with NAFLD risk in some developed countries, like the United States, while potential gender-specific effects were found among the Chinese population. **Conclusions:** The current meta-analysis suggests that the association between educational attainment and NAFLD is complex and context-dependent, and it may vary across different countries and types of sex.

## 1. Introduction

Non-alcoholic fatty liver disease (NAFLD) is a metabolic disorder characterized by hepatic fat accumulation without significant alcohol consumption or other identifiable liver-damaging factors, and affects approximately 30% of the global population and may progress to severe complications, including hepatocellular carcinoma, posing substantial healthcare burdens [1,2]. Contemporary etiological research has established NAFLD development as a result of complex interactions between lifestyle factors [3,4], genetic susceptibility [5], demographic characteristics [6,7], and multiple metabolic or psychological comorbidities [8,9], forming a dynamic pathophysiological network.

Growing evidence underscores the pivotal role of social determinants of health in shaping NAFLD epidemiology, profoundly influencing disease distribution and population-level burden [10]. Population-based studies in the US demonstrated that higher income levels are associated with lower liver disease prevalence [11] and reduced liver-related mortality [12], likely attributable to economic barriers limiting healthcare access, healthy living conditions, and nutritional adequacy in low-income groups. Yet some Chinese studies found high income without corresponding higher education and health awareness may paradoxically increase NAFLD risk [13], highlighting the importance of education. Occupational exposures, such as prolonged work hours, shift work, and sedentary occupations, were found independently linked to metabolic dysfunction, increased NAFLD incidence [14,15], and elevated all-cause mortality in male populations [16]. These social determinants exert sustained influence through direct biological mechanisms (e.g., chronic stress-induced metabolic dysregulation) and indirect behavioral pathways (e.g., dietary choices, physical inactivity), ultimately serving as essential proxies for broader disease risk constellations and systematically integrating into contemporary risk prediction models to identify vulnerable populations [11,17,18], moving beyond traditional biomedical risk factors to a certain extent.

As a fundamental component of socioeconomic status, educational attainment offers distinct advantages in epidemiological research due to its stability, standardized measurement, and cross-population comparability [19]. Existing studies have identified plausible biological and social mechanisms linking educational attainment to NAFLD pathogenesis [3,20,21,22,23], offering new perspectives on the social determinants of disease. However, these studies suffer from notable limitations: inconsistent findings, insufficient power for subgroup analyses, and failure to account for potential confounders. Therefore, this study aims to conduct a systematic review and meta-analysis to investigate the actual relationship and potential influencing factors between educational attainment and NAFLD.

## 2. Materials and Methods

### 2.1. Study Design

This systematic review and meta-analysis followed the PRISMA guidelines [24]. The study protocol was registered on the International Prospective Register of Systematic Reviews (registration number: CRD42024598095). No similar systematic review protocol exists in the current literature.

### 2.2. Search Strategy

A comprehensive literature search was conducted in PubMed, Web of Science, and Scopus from inception to 31 December 2024, without language restrictions. The search integrated keywords and Medical Subject Headings (MeSH) terms related to “education” and “NAFLD” using Boolean operators (Supplementary Material S1). Two researchers independently screened titles/abstracts against eligibility criteria, reviewed full texts of potential studies for final inclusion, and resolved disagreements via discussion or a third researcher.

### 2.3. Eligibility Criteria

#### 2.3.1. Inclusion Criteria

Studies were included if they met all of the following criteria: (1) Were observational studies, including cross-sectional, cohort, or case-control designs; (2) Reported baseline data with quantifiable metrics of the association between educational attainment and NAFLD status; (3) Included NAFLD individuals and corresponding healthy individuals without liver disease; (4) Classified educational attainment as follows: more than high school, high school education, and less than high school, or data convertible to this classification.

#### 2.3.2. Exclusion Criteria

Studies were excluded if they met any of the following criteria: (1) Involved non-human subjects; (2) Focused on populations with competing liver pathologies (e.g., viral hepatitis or drug-induced liver injury); (3) Were secondary analyses or case reports (4) Were retracted or had unavailable full texts.

### 2.4. Data Collection

Two independent researchers extracted data in duplicate using standardized forms and discrepancies were resolved via discussion with a third reviewer. The extracted data included: (1) Study characteristics (first author’s name, year of publication, Digital Object Identifier (DOI), country of origin); (2) Demographic and clinical data analyzed in the study (sample size, number of NAFLD populations and the corresponding healthy individuals categorized by educational attainment); (3) Other details (participant sex, age range, population source, NAFLD diagnostic criteria).

### 2.5. Data Processing

Each stratum was treated as an individual data entry when primary studies conducted stratified analyses based on certain characteristics (e.g., sex). Duplicate and covered data were removed by comparing them with several factors (e.g., population sources, observation timeframes, sample sizes, sampling methods). These approaches enable a more comprehensive and accurate data analysis within each subgroup. Unanimous agreement among the three reviewers was imperative for all decisions.

### 2.6. Risk of Bias Assessment

Two independent researchers assessed study quality using the AHRQ (Agency for Healthcare Research and Quality) checklist, excluding item #11 (follow-up assessment, irrelevant to cross-sectional designs). Ten items were evaluated, including study design appropriateness, sample representativeness, data collection reliability, risk-of-bias domains, and other relevant details. Studies received 1 point for each “yes” response (satisfactory addressing of an item) and 0 points for “no” or “unclear” responses. The total score was used to evaluate study quality: 0–2 indicates low quality, 3–6 indicates moderate quality, and 7–10 indicates high quality with low risk of bias. Any disagreements were resolved through re-examination and discussion, or by consulting a third researcher.

### 2.7. Data Analysis

All statistical analyses were performed using Review Manager 5.4 (The Cochrane Collaboration). For primary analysis, we calculated pooled odds ratios (ORs) with 95% confidence intervals (CIs), defining statistical significance as *p*  <  0.05. Heterogeneity was assessed using the I^2^ statistic with Mantel-Haenszel tests. An I^2^ value ≤50% indicated acceptable heterogeneity, and a fixed-effects model was used. An I^2^ value >50% indicated considerable heterogeneity, and a random-effects model was applied. For analyses including ≥3 studies, subgroup analyses were conducted to identify potential sources of heterogeneity. For analyses including ≥5 studies, “leave-one-out” sensitivity analyses were performed by sequentially omitting each study to recalculate pooled estimates and evaluate effect size stability. Publication bias was evaluated using a combination of graphical and statistical methods. Funnel plots were generated using Review Manager 5.4, and the symmetry indicated a low likelihood of publication bias. Egger’s regression test was performed using Stata version 11 (StataCorp LP, College Station, TX, USA) to quantify funnel plot asymmetry by evaluating the deviation of the intercept from zero. This assessment was interpreted in conjunction with effect sizes and heterogeneity metrics to evaluate evidence reliability comprehensively.

## 3. Results

### 3.1. Search Results

**Figure 1:**
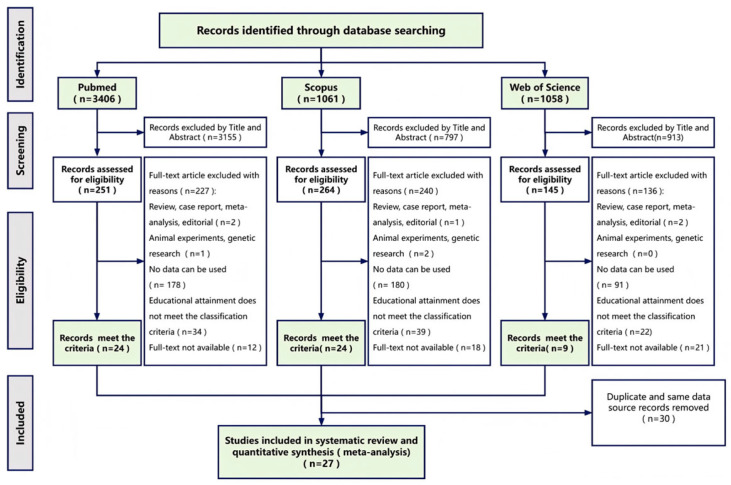
Flow diagram of the study selection process.

A comprehensive systematic search was conducted across three major databases (PubMed: 3403; Web of Science: 1058; Scopus: 1061) using the predefined search strategy, yielding 5522 potentially relevant articles. After literature screening, duplicate removing, quality evaluating and data processing, 27 studies were retained, yielding a total of 34 datasets included in the final meta-analysis (Figure 1).

### 3.2. Study Characteristics

**Figure 2:**
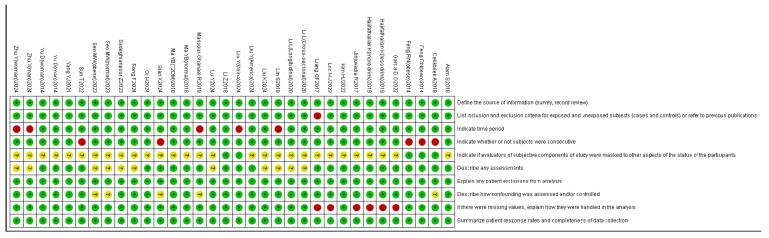
Quality assessment of the literature [10,23,25,26,27,28,29,30,31,32,33,34,35,36,37,38,39,40,41,42,43,44,45,46,47,48,49].

The meta-analysis included 27 studies from six countries, with a total pooled sample of 446,312 participants (93,116 NAFLD and 353,196 healthy individuals). Geographically, the studies included 11 from China [23,25,26,27,28,29,30,31,32,33,34], 8 from the United States [35,36,37,38,39,40,41,42], 4 from South Korea [43,44,45,46], and 4 from three other countries, including Iran [10,47,48,49]. Methodologically, the studies comprised 24 cross-sectional studies, 2 case-control studies, and 1 prospective cohort study, all compatible with the modified AHRQ quality assessment tool. For NAFLD diagnosis, 26 studies used objective methods (ultrasonography or validated indices like FLI and HIS), while one relied on a physical examination report, which may introduce detection bias. Quality assessment via the adapted AHRQ checklist showed uniformly high methodological quality, with none falling into the moderate or low categories. There were several notable limitations, including insufficient distinction between subjective and objective assessments, suboptimal handling of missing data, and inadequate quality control for primary outcome measurement (Figure 2). Study-specific details are provided in Table 1.

### 3.3. Findings About Associations Between Educational Attainment and NAFLD

**Figure 3:**
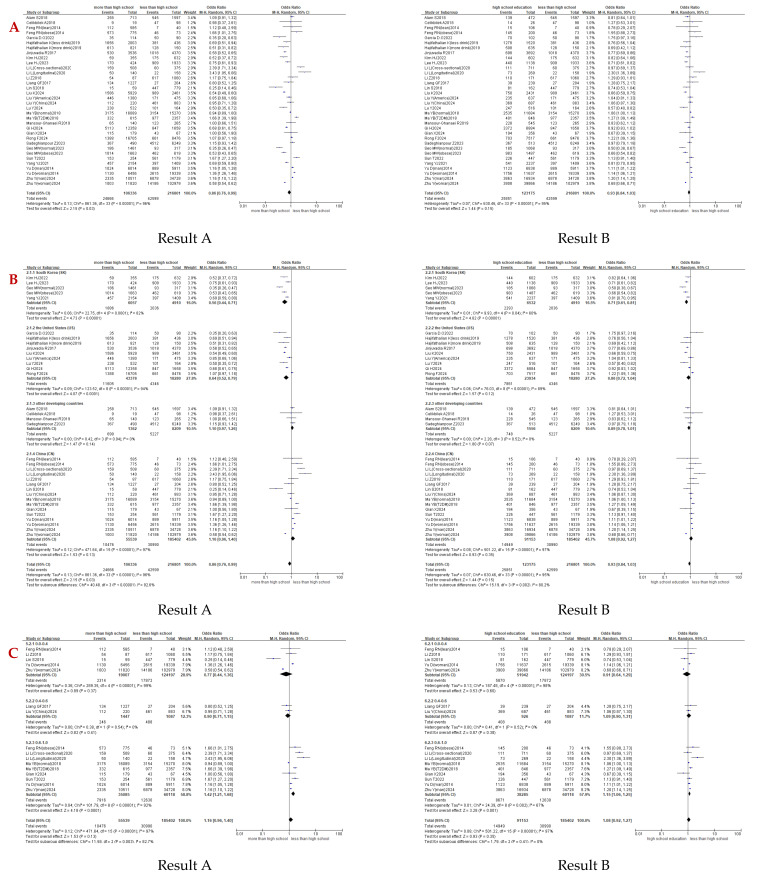

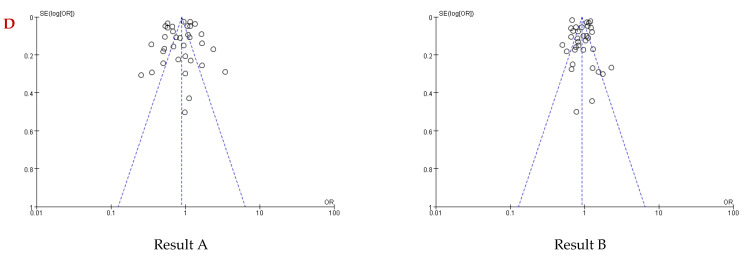
In the following content, two results are presented: Result A is the comparison between “more-than-high-school” and “less-than-high-school”. Result B is the comparison between “high-school-education” and “less-than-high-school” [10,23,25,26,27,28,29,30,31,32,33,34,35,36,37,38,39,40,41,42,43,44,45,46,47,48,49]. (**A**): Associations Between Educational Attainment and NAFLD [10,23,25,26,27,28,29,30,31,32,33,34,35,36,37,38,39,40,41,42,43,44,45,46,47,48,49]. (**B**): Subgroup Analysis by Country [10,23,25,26,27,28,29,30,31,32,33,34,35,36,37,38,39,40,41,42,43,44,45,46,47,48,49]. (**C**): Subgroup Analysis by Male Proportion [23,25,26,27,28,29,30,31,32,33,34]. (**D**): Publication Bias Assessment.

The meta-analysis revealed substantial heterogeneity across studies (I^2^ > 50%), justifying the use of random-effects models. More-than-high-school was inversely associated with NAFLD risk compared to the reference group (less-than-high-school) (OR = 0.86, 95%CI: 0.76 to 0.99; I^2^ = 96%, *p* = 0.03). High-school-education showed no statistically significant association (OR = 0.93, 95%CI: 0.84 to 1.03; I^2^ = 95%, *p* = 0.15) (Figure 3A). High heterogeneity suggested that the interpretation of the OR value may lack accuracy.

### 3.4. Subgroup Analyses of the Association Between Educational Attainment and NAFLD

#### 3.4.1. Subgroup Analysis by Country

A stratified analysis was conducted across four national subgroups: China (CN), the United States (US), South Korea (SK), and other developing countries (the latter combining nations with <3 eligible studies). The analysis revealed significant heterogeneity in the association across subgroups, and substantial internal heterogeneity was observed in all subgroups except developing countries. When comparing more-than-high-school vs. less-than-high-school groups, a distinct geographic gradient emerged. Inverse associations were found in South Korea and the United States, while non-significant positive associations were observed in other developing countries and China (OR_SK_ = 0.56, 95%CI = 0.44 to 0.71; OR_US =_ 0.64, 95%CI = 0.52 to 0.79; OR_developing_ = 1.10, 95%CI = 0.97 to 1.26; OR_CN_ = 1.16, 95%CI = 0.96 to 1.40; I^2^_subgroup_ = 92.6%, *p* < 0.00001). A similar but less pronounced trend was also observed in high-school-education vs. less-than-high-school comparisons (OR_SK_ = 0.71, 95%CI = 0.61 to 0.81; OR_US_ = 0.86, 95%CI = 0.72 to 1.04; OR_developing_ = 0.89, 95%CI = 0.78 to 1.01; OR_CN_ = 1.08, 95%CI = 0.92 to 1.27; I^2^_subgroup_ = 80.2%, *p* = 0.002). These trends highlighted the impact of national factors on the association and underscored the methodological necessity of national subgrouping, as aggregate analyses might obscure context-dependent patterns (Figure 3B).

#### 3.4.2. Subgroup Analysis by Age Range

Studies with sufficient demographic data were stratified into three age subgroups (Q1: ≤40 years; Q2: 40–50 years; Q3: ≥50 years) using Wan’s standardized categorization method [50], based on calculated mean age values, to investigate potential age-dependent effects on the association. Although this stratification modestly reduced within-group heterogeneity, significant between-group differences emerged only in the Chinese subgroup in the more-than-high-school vs. less-than-high-school comparison. However, this finding likely reflected the exclusive inclusion of male participants in China’s Q1 subgroup rather than a true age effect [26]. No statistical significance and consistent age-related trends were observed in other comparisons. These results indicated that while age may contribute to some degree of heterogeneity, its effects were likely relatively limited, warranting cautious interpretation of age-stratified analyses (Supplementary Material S2, Part 1).

#### 3.4.3. Subgroup Analysis by Male Proportion

Studies were stratified into three subgroups according to male proportion (0.0–0.4, 0.4–0.6, 0.6–1.0) to evaluate potential gender-specific effects. The analysis revealed that, in China, as male proportion increased, the association tended to shift from inverse to positive (more-than-high-school vs. less-than-high-school: OR_0.0–0.4_ = 0.77, 95%CI = 0.44 to 1.36; OR_0.4–0.6_ = 0.90, 95%CI = 0.71 to 1.15; OR_0.6–1.0_ = 1.42, 95%CI = 1.21 to 1.68; I^2^ subgroup = 82.7%, *p* = 0.00; high-school-education vs. less-than-high-school: OR_0.0–0.4_ = 0.91, 95%CI = 0.64 to 1.29; OR_0.4–0.6_ = 1.09, 95%CI = 0.90 to 1.31; OR_0.6–1.0_ = 1.15, 95%CI = 1.06 to 1.25; I^2^subgroup = 0%, *p* = 0.41). By contrast, this trend was absent in the American population. These findings demonstrated a potential gender-specific effect, which was only observed in China (Figure 3C).

### 3.5. Sensitivity Analysis

Sensitivity analyses identified several influential studies [23,29,30,40,41,42] as major sources of subgroup heterogeneity and effect size variation. Excluding these studies led to a marked reduction in heterogeneity of the subgroup analysis conducted by country, and induced modest changes in pooled effect estimates. Crucially, the core qualitative conclusions persisted, including significant differences across national subgroups and potential gender-specific effects among the Chinese population, which means that the primary study conclusions remained robust and reliable (Supplementary Material S2, Part 2).

### 3.6. Publication Bias Assessment

The funnel plot and Egger’s test (P_1_ = 0.572; P_2_ = 0.799) suggested minimal publication bias (Figure 3D). The predominantly cross-sectional nature of the included studies and the indirect relationship between NAFLD outcomes and educational attainment, which were typically collected as demographic covariates, provided additional contextual evidence for low publication bias risk in this study.

## 4. Discussion

This systematic review and meta-analysis elucidated the complex association between educational attainment and NAFLD risk, demonstrating significant variations across geographical regions and gender groups.

Subgroup analyses revealed that in developed nations, such as the United States and South Korea from this study, and several European countries from other studies (e.g., Russia, Finland, the UK, the Netherlands, and Switzerland), higher education was inversely associated with NAFLD risk, while higher education was positively associated with NAFLD risk in developing nations, such as China, Iran, and Bangladesh from this study, and Sri Lanka reported elsewhere [20,51,52,53,54,55,56]. As one study reported a similar phenomenon in cardiovascular disease risk [57], we hypothesized that national economic development may be one of the influencing factors, with potential mechanisms outlined below.

Firstly, in developing countries, individuals with higher education may adopt certain unhealthy lifestyles, increasing their susceptibility to NAFLD. Although higher education typically promotes healthier lifestyles, including balanced nutrition, regular physical activity, and good sleep hygiene [3,4,58,59,60], its inverse association with NAFLD risk varies across populations. For example, studies have shown that in southern China, highly educated individuals face an elevated risk of NAFLD, potentially due to poor sleep quality or excessive daytime napping [61,62]. Meanwhile, evidence from multiple studies indicates that higher educational attainment is frequently associated with sedentary occupations characterized by prolonged sitting, reduced physical activity, and heightened psychological stress [14,63,64], all of which are collectively linked to an increased risk of NAFLD development. These inconsistent patterns help to elucidate the complex association between educational attainment and NAFLD risk.

Secondly, the combination of food insecurity in childhood and unhealthy dietary choices in adulthood offers another plausible explanation. Several studies have demonstrated that childhood food insecurity, coupled with associated economic deprivation, food scarcity, and malnutrition, significantly elevates the risk of chronic liver disease in adulthood, including NAFLD [65,66,67]. In developing regions, highly educated adults tend to consume more food and opt for energy-dense, ultra-processed items if their predicaments in childhood are changed, all of which dietary choices are well-established dietary risk factors for NAFLD development, owing to their higher socioeconomic status and greater food accessibility [67,68,69]. Scholars term this compensatory pattern “catch-up growth”, characterized by adult metabolic disorders stemming from early-life nutritional constraints, such as food insecurity, low birth weight, and childhood illnesses [70,71,72,73]. Given regional economic disparities, food insecurity is more widespread in developing countries, which may account for the elevated NAFLD risk among their educated demographics.

Thirdly, genetic polymorphism may be another possible reason for the regional and potential gender differences in the education-NAFLD relationship. Two Mendelian randomization studies using GWAS and MR-Base datasets explore this relationship and consider genetic factors as primary regulators, and both educational attainment and NAFLD risk as interrelated outcomes of shared genetic architecture [74,75]. This theory provides a biological basis for regional disparities and gender-specific effects in the association, revealing additional pathways linking education to NAFLD.

Regarding gender-specific disparities in the Chinese population, prior studies have posited that these may stem from gender-based differences in healthy lifestyle tendencies [23,33]. It is also noteworthy that the difference between men and women in the genetic polymorphisms, along with other potential factors (e.g., dietary preference, career choices, social division of labor), might also explain the observed disparities in this association.

However, population-level findings may not fully reflect individual heterogeneity, suggesting caution is needed when examining gender-specific associations between educational attainment and NAFLD risk.

It is important to clarify that educational attainment does not directly influence the development of NAFLD. Instead, what matters are the changes in intermediate factors associated with different levels of education, such as diet, lifestyle, healthcare quality, and physical activity. Educational attainment may, to some extent, reflect the combined effects of these intermediate factors on the disease. This study indicates that developing regions require more comprehensive interventions that simultaneously address education, healthcare system enhancement, dietary pattern improvement, and physical activity infrastructure improvements to achieve meaningful NAFLD risk reduction. For highly educated men in China, increasing physical activity, reducing the frequency of eating out, adopting a light diet, decreasing alcohol consumption and smoking, relieving mental stress, and ensuring adequate sleep are all likely to bring significant health benefits. Additionally, based on employees’ specific work types and energy expenditure, companies could design low-fat, low-salt, reduced-calorie meals in their canteens, or arrange appropriate activity sessions or midday breaks during work hours to help their employees prevent chronic diseases such as NAFLD. At the same time, we should also pay attention to groups with low educational attainment, as their socioeconomic disadvantage usually means a shortage of medical resources. When incorporating educational attainment into population-based chronic liver disease prediction models, it is crucial to comprehensively consider how local socioeconomic conditions and gender influence the association.

To the best of our knowledge, this is the first comprehensive meta-analysis to quantify the influence of socioeconomic factors and gender on the association between educational attainment and NAFLD risk. However, this study is constrained by several major methodological limitations. First, significant residual heterogeneity remained in some comparative analyses, primarily attributed to disparities in sampling methods, participant traits (e.g., BMI, alcohol consumption), and variations in NAFLD diagnostic methods and non-standardized educational attainment categories across studies, as well as some unmeasured but potentially influential factors (e.g., cultural differences). Second, while sensitivity analyses supported our qualitative conclusions, quantitative results lacked stability, hampering the exploration of interactions between influencing factors and limiting the ability to elucidate the mechanisms underlying the education-NAFLD relationship. Third, the data mainly came from cross-sectional studies in China and the United States, which not only resulted in insufficient causal inference and exploration of factors influencing the association but also led to inadequate representation of low-income countries, both of which limited the generalizability of our conclusions. Thus, future prospective cohort studies are needed to further explore the mechanisms underlying the association between educational attainment and NAFLD and related influencing factors, while diverse-population studies can help elucidate the true nature of this association, specifically in low-income countries, and enhance the generalizability of the results.

## 5. Conclusions

This study aims to explore the association between educational attainment and NAFLD risk, and to investigate potential influencing factors. Our findings advance evidence-based identification of socially vulnerable NAFLD populations and strengthen the implementation of social medicine strategies in chronic disease prevention.

## Figures and Tables

**Table 1:** Characteristics of included studies.

Author	Time	Region	Method	Events	Total	Age	Sex	Source	Diagnostic
Jinjuvadia, R. [35]	2017	America	Cross	2246	11,598	42.1	0.4–0.6	NHANES III	ultrasound
Hajifathalian, K. (less drink) [38]	2019	America	Cross	3315	3959	48.4	0.4–0.6	NHANES	Index
Hajifathalian, K. (more drink) [38]	2019	America	Cross	1249	1606	47.2	0.6–1.0	NHANES	Index
Garcia, D.O. [37]	2022	America	Cross	155	306	44.5	0.0–0.4	Arizona Liver Health	ultrasound
Liu, K. [39]	2024	America	Cross	3325	10,821	N/A	0.4–0.6	NHANES	Index
Liu, Y. (America) [36]	2024	America	Cross	852	2492	51.0	0.4–0.6	NHANES	ultrasound
Lu, Y. [40]	2024	America	Cross	586	1212	55.3	0.0–0.4	ARIC	Index
Qi, H. [41]	2024	America	Cross	9332	20,900	48.1	0.4–0.6	NHANES	Index
Rong, F. [42]	2024	America	Cross	2752	32,698	N/A	0.4–0.6	NHANES	Index
Alam, S. [47]	2018	Bangladesh	Cross	942	2782	34.2	0.6–1.0	Dhaka City	ultrasound
Feng, R.N. (lean) [27]	2014	China	Cross	134	731	44.1	0.0–0.4	Second Affiliated Hospital of Harbin	ultrasound
Feng, R.N. (obese) [27]	2014	China	Cross	764	1048	46.9	0.6–1.0	Second Affiliated Hospital of Harbin	ultrasound
Yu, D. (man) [34]	2014	China	Cross	3036	18,763	53.7	0.6–1.0	SWHS&SMHS	ultrasound
Yu, D. (woman) [34]	2014	China	Cross	5501	37,432	51.4	0.0–0.4	SWHS&SMHS	ultrasound
Liang, Q.F. [25]	2017	China	Cross	200	1670	40.7	0.4–0.6	Hebei Province	physical examination
Li, Z. [29]	2018	China	Cross	781	1318	53.4	0.0–0.4	Lianqian community	ultrasound
Lin, S. [30]	2018	China	Cross	543	1000	51.8	0.0–0.4	Four communities in Urumqi Province	ultrasound
Ma, Y.B. (normal) [26]	2018	China	Cross	8864	43,043	45.8	0.6–1.0	Jinchang Cohort	ultrasound
Ma, Y.B. (T2DM) [26]	2018	China	Cross	1710	3818	58.8	0.6–1.0	Jinchang Cohort	ultrasound
Li, L. (Cross-sectional) [28]	2020	China	Cross	330	1594	35.6	0.6–1.0	FAMHES	ultrasound
Li, L. (Longitudinal) [28]	2020	China	Longitudinal	145	567	37.5	0.6–1.0	FAMHES	ultrasound
Sun, T. [33]	2022	China	Case-Control	940	1880	54.2	0.6–1.0	Tongji-Ezhou Study	ultrasound
Liu, Y. (China) [31]	2024	China	Cross	942	1790	65.2	0.4–0.6	Dongfeng-Tongji cohort	ultrasound
Qian, X. [32]	2024	China	Cross	352	602	49.0	0.6–1.0	an enterprise health lodge	ultrasound
Zhu, Y. (man) [23]	2024	China	Cross	13,076	62,173	58.9	0.6–1.0	REACTION	Index
Zhu, Y. (woman) [23]	2024	China	Cross	19,097	154,665	56.9	0.0–0.4	REACTION	Index
Mansour-Ghanaei, R. [49]	2019	Iran	Cross	416	950	N/A	0.6–1.0	PERSIAN Guilan Cohort Study	ultrasound
Sadeghianpour, Z. [10]	2023	Iran	Cross	5246	7252	48.7	0.0–0.4	Hoveyzeh Cohort Study(HCS)	Index
Yang, Y.J. [46]	2021	Korea	Cross	1395	5800	46.2	0.4–0.6	KoNEHS(II)	Index
Kim, H.J. [43]	2022	Korea	Cross	378	1589	55.0	0.4–0.6	KNHANES	Index
Lee, H.J. [44]	2023	Korea	Cross	1519	3495	55.5	0.4–0.6	Ansan–Ansung	Index
Seo, M.W. (normal) [45]	2023	Korea	Cross	464	2846	43.7	0.0–0.4	KHANES	Index
Seo, M.W. (obese) [45]	2023	Korea	Cross	2459	3769	46.8	0.4–0.6	KHANES	Index
Celikbilek, A. [48]	2018	Turkey	Case-Control	70	143	45.4	0.0–0.4	Bozok University	ultrasound

N/A, not applicable.

## Data Availability

The original contributions presented in this study are included in the article/Supplementary Material. Further inquiries can be directed to the corresponding author.

## Supplementary Materials

The following supporting information can be downloaded at: https://www.mdpi.com/article/10.3390/healthcare14091197/s1, Supplementary Material S1: Full search strategy for PubMed, Scopus and Web of Science Core Collection electronic databases and simplified version of main search terms; Supplementary Material S2: Characteristics of included studies; Supplementary Material S3: Results, Sensitivity Analysis, and Publication Bias.
