# Supplementary material for: The Association Between Educational Attainment and Non-Alcoholic Fatty Liver Disease: A Systematic Review and Meta-Analysis of Observational Studies
Source: Healthcare (Basel). 2026 Apr 29;14(9):1197. doi: 10.3390/healthcare14091197 (PMC13164538; doi:10.3390/healthcare14091197)
Supplement: Supplementary file 1 [file healthcare-14-01197-s001.zip › Supplementary Material S1.pdf]

Supplementary Material S1 - Full search strategy for PubMed, Scopus and Web of Science Core Collection electronic databases and simplified version of main search terms

Database	Search terms
PubMed [All Fields]	#1 Search:<br><br>Non-alcoholic Fatty Liver Disease [MeSH Terms]<br><br>#2 Search:<br><br>(NAFLD) OR (Non alcoholic Fatty Liver Disease) OR (Fatty Liver, Nonalcoholic) OR (Fatty Livers, Nonalcoholic) OR (Liver, Nonalcoholic Fatty) OR (Livers, Nonalcoholic Fatty) OR (Nonalcoholic Fatty Liver) OR (Nonalcoholic Fatty Livers) OR (Nonalcoholic Fatty Liver Disease) OR (Nonalcoholic Steatohepatitis) OR (Nonalcoholic Steatohepatitides) OR (Steatohepatitides, Nonalcoholic) OR (Steatohepatitis, Nonalcoholic)<br><br>#3 Search: #1 OR #2<br><br>#4 Search:<br><br>Education Status [MeSH Terms]<br><br>#5 Search: (education) OR (educational level) OR (education levels) OR (level of education) OR (educational status) OR (education status) OR (educational achievement) OR (academic achievement) OR (academic qualifications) OR (education degree) OR (achievement educational) OR (educational attainment)<br><br>#6 Search: #4 OR #5<br><br>#7 Search: #3 AND #6AND<br><br>From library building- 2024/12/31
Scopus	(TITLE-ABS-KEY ((NAFLD) OR (Non alcoholic Fatty Liver Disease) OR (Fatty Liver, Nonalcoholic) OR (Fatty Livers, Nonalcoholic) OR (Liver, Nonalcoholic Fatty) OR (Livers, Nonalcoholic Fatty) OR (Nonalcoholic Fatty Liver) OR (Nonalcoholic Fatty Livers) OR (Nonalcoholic Fatty Liver Disease) OR (Nonalcoholic Steatohepatitis) OR (Nonalcoholic Steatohepatitides) OR (Steatohepatitides, Nonalcoholic) OR (Steatohepatitis, Nonalcoholic)) AND TITLE-ABS-KEY ((education) OR (educational level) OR (education levels) OR (level of education) OR (educational status) OR

	(education status) OR (educational achievement) OR (academic achievement) OR (academic qualifications) OR (education degree) OR (achievement educational) OR (educational attainment))<br><br>From library building- 2024/12/31
Web of Science	((TS=((NAFLD) OR (Non alcoholic Fatty Liver Disease) OR (Fatty Liver, Nonalcoholic) OR (Fatty Livers, Nonalcoholic) OR (Liver, Nonalcoholic Fatty) OR (Livers, Nonalcoholic Fatty) OR (Nonalcoholic Fatty Liver) OR (Nonalcoholic Fatty Livers) OR (Nonalcoholic Fatty Liver Disease) OR (Nonalcoholic Steatohepatitis) OR (Nonalcoholic Steatohepatitides) OR (Steatohepatitides, Nonalcoholic) OR (Steatohepatitis, Nonalcoholic)) AND TS=((education) OR (educational level) OR (education levels) OR (level of education) OR (educational status) OR (education status) OR (educational achievement) OR (academic achievement) OR (academic qualifications) OR (education degree) OR (achievement educational) OR (educational attainment))<br><br>From library building- 2024/12/31
