# Supplementary material for: The Association Between Educational Attainment and Non-Alcoholic Fatty Liver Disease: A Systematic Review and Meta-Analysis of Observational Studies
Source: Healthcare (Basel). 2026 Apr 29;14(9):1197. doi: 10.3390/healthcare14091197 (PMC13164538; doi:10.3390/healthcare14091197)
Supplement: Supplementary file 1 [file healthcare-14-01197-s001.zip › Supplementary Material S2.pdf]

Supplementary Material S2-Results, Sensitivity Analysis, and Publication Bias

In the following content, two results are presented:

Result A is the comparison between “more-than-high-school” and “less-than-high-school”.

Result B is the comparison between “high-school-education” and “less-than-high-school”.

## Part 1-Supgroup Analysis By Age

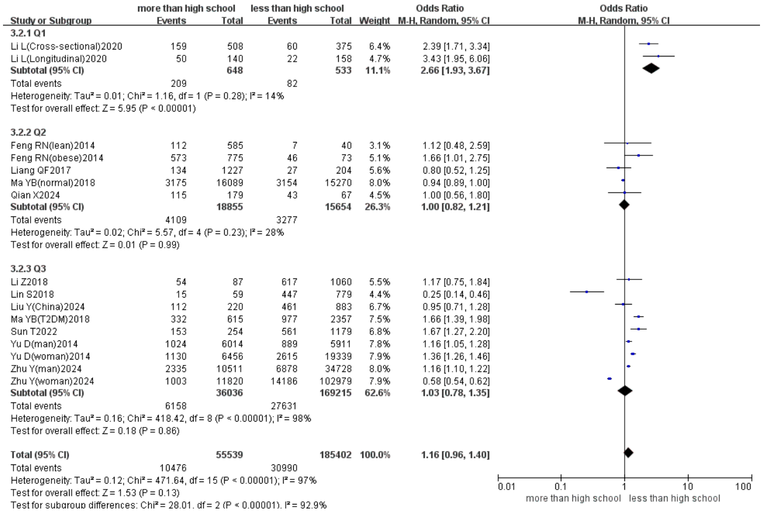


Result A

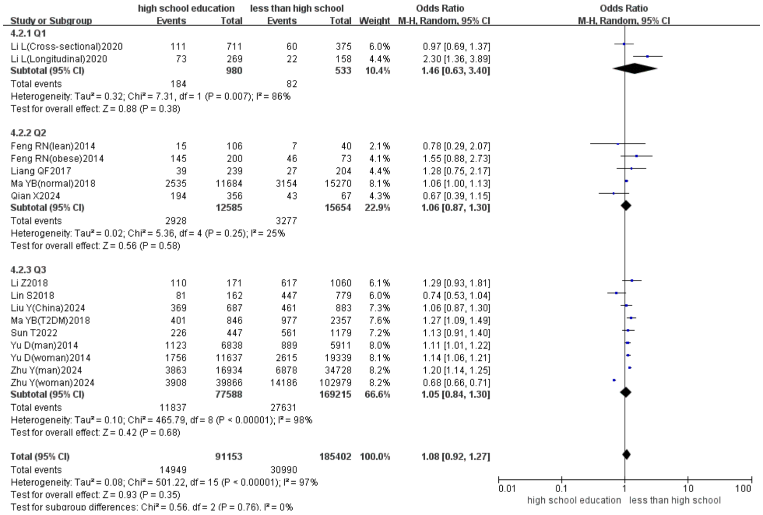


Result B

Figure S1.1: Subgroup analysis by age in China [23,25–34].

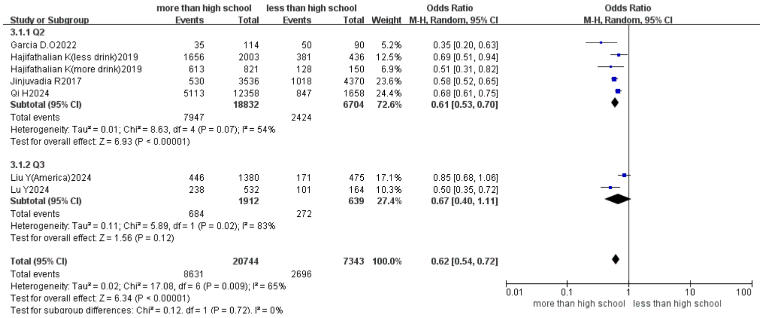


Result A

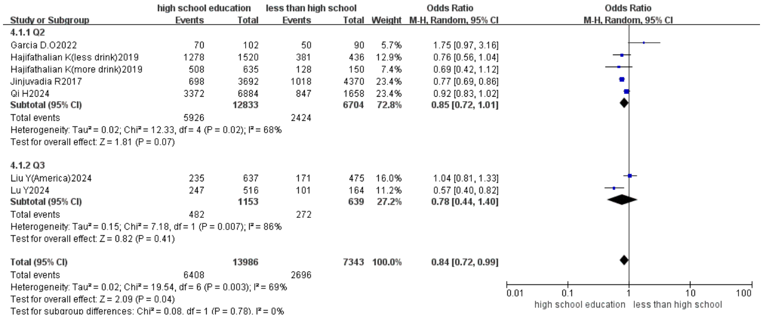


Result B

Figure S1.2: Subgroup analysis by age in the United States [35–38,40,41].

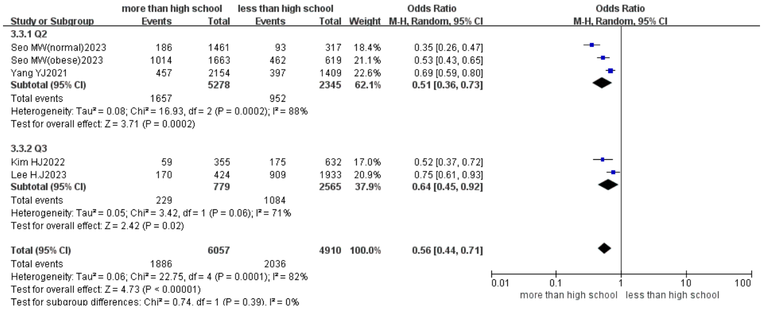


Result A

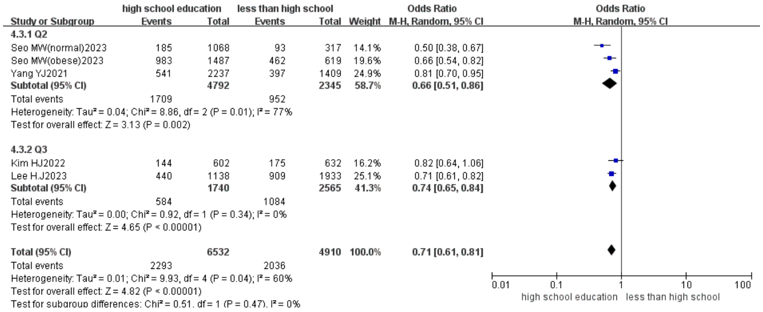


Result B

Figure S1.3: Subgroup analysis by age in South Korea [43–46].

Part 2-Sensitive Analysis

| Study or Subgroup | more than high school Events | Total | less than high school Events | Total | Weight | Odds Ratio M-H, Random, 95% CI |
|---|---|---|---|---|---|---|
| **2.1.1 South Korea (SK)** | | | | | | |
| Kim HJ2022 | 59 | 355 | 175 | 632 | 4.0% | 0.52 [0.37, 0.72] |
| Lee H.J2023 | 170 | 424 | 909 | 1933 | 4.3% | 0.75 [0.61, 0.93] |
| Seo MW(normal)2023 | 186 | 1461 | 93 | 317 | 4.1% | 0.35 [0.26, 0.47] |
| Seo MW(obese)2023 | 1014 | 1663 | 462 | 619 | 4.3% | 0.53 [0.43, 0.65] |
| Yang YJ2021 | 457 | 2154 | 397 | 1409 | 4.4% | 0.69 [0.59, 0.80] |
| **Subtotal (95% CI)** | | **6057** | | **4910** | **21.2%** | **0.56 [0.44, 0.71]** |
| Total events | 1886 | | 2036 | | | |
| Heterogeneity: Tau² = 0.06; Chi² = 22.75, df = 4 (P = 0.0001); I² = 82% | | | | | | |
| Test for overall effect: Z = 4.73 (P < 0.00001) | | | | | | |
| **2.1.2 the United States (US)** | | | | | | |
| Garcia D.O2022 | 35 | 114 | 50 | 90 | 3.2% | 0.35 [0.20, 0.63] |
| Hajifathalian K(less drink)2019 | 1656 | 2003 | 381 | 436 | 4.1% | 0.69 [0.51, 0.94] |
| Hajifathalian K(more drink)2019 | 613 | 821 | 128 | 150 | 3.5% | 0.51 [0.31, 0.82] |
| Jinjuvadia R2017 | 530 | 3536 | 1018 | 4370 | 4.5% | 0.58 [0.52, 0.65] |
| Liu K2024 | 1586 | 5929 | 989 | 2461 | 4.5% | 0.54 [0.49, 0.60] |
| Liu Y(America)2024 | 446 | 1380 | 171 | 475 | 0.0% | 0.85 [0.68, 1.06] |
| Lu Y2024 | 238 | 532 | 101 | 164 | 3.9% | 0.50 [0.35, 0.72] |
| Qi H2024 | 5113 | 12358 | 847 | 1658 | 0.0% | 0.68 [0.61, 0.75] |
| Rong F2024 | 1388 | 16705 | 661 | 8476 | 0.0% | 1.07 [0.97, 1.18] |
| **Subtotal (95% CI)** | | **12935** | | **7671** | **23.7%** | **0.56 [0.52, 0.60]** |
| Total events | 4658 | | 2667 | | | |
| Heterogeneity: Tau² = 0.00; Chi² = 5.39, df = 5 (P = 0.37); I² = 7% | | | | | | |
| Test for overall effect: Z = 14.56 (P < 0.00001) | | | | | | |
| **2.1.3 other developing countries** | | | | | | |
| Alam S2018 | 258 | 713 | 545 | 1597 | 4.4% | 1.09 [0.91, 1.32] |
| Celikbilek A2018 | 9 | 19 | 47 | 98 | 2.0% | 0.98 [0.37, 2.61] |
| Mansour-Ghanaei R2019 | 65 | 140 | 123 | 265 | 3.7% | 1.00 [0.66, 1.51] |
| Sadeghianpour Z2023 | 367 | 490 | 4512 | 6249 | 4.3% | 1.15 [0.93, 1.42] |
| **Subtotal (95% CI)** | | **1362** | | **8209** | **14.4%** | **1.10 [0.97, 1.26]** |
| Total events | 699 | | 5227 | | | |
| Heterogeneity: Tau² = 0.00; Chi² = 0.42, df = 3 (P = 0.94); I² = 0% | | | | | | |
| Test for overall effect: Z = 1.47 (P = 0.14) | | | | | | |
| **2.1.4 China (CN)** | | | | | | |
| Feng RN(lean)2014 | 112 | 585 | 7 | 40 | 2.4% | 1.12 [0.48, 2.59] |
| Feng RN(obese)2014 | 573 | 775 | 46 | 73 | 3.4% | 1.66 [1.01, 2.75] |
| Li L(Cross-sectional)2020 | 159 | 508 | 60 | 375 | 4.0% | 2.39 [1.71, 3.34] |
| Li L(Longitudinal)2020 | 50 | 140 | 22 | 158 | 3.2% | 3.43 [1.95, 6.06] |
| Li Z2018 | 54 | 87 | 617 | 1060 | 3.6% | 1.17 [0.75, 1.84] |
| Liang QF2017 | 134 | 1227 | 27 | 204 | 3.6% | 0.80 [0.52, 1.25] |
| Lin S2018 | 15 | 59 | 447 | 779 | 0.0% | 0.25 [0.14, 0.46] |
| Liu Y(China)2024 | 112 | 220 | 461 | 883 | 4.1% | 0.95 [0.71, 1.28] |
| Ma YB(normal)2018 | 3175 | 16089 | 3154 | 15270 | 0.0% | 0.94 [0.89, 1.00] |
| Ma YB(T2DM)2018 | 332 | 615 | 977 | 2357 | 0.0% | 1.66 [1.39, 1.98] |
| Qian X2024 | 115 | 179 | 43 | 67 | 3.2% | 1.00 [0.56, 1.80] |
| Sun T2022 | 153 | 254 | 561 | 1179 | 4.1% | 1.67 [1.27, 2.20] |
| Yu D(man)2014 | 1024 | 6014 | 889 | 5911 | 4.5% | 1.16 [1.05, 1.28] |
| Yu D(woman)2014 | 1130 | 6456 | 2615 | 19339 | 4.5% | 1.36 [1.26, 1.46] |
| Zhu Y(man)2024 | 2335 | 10511 | 6878 | 34728 | 0.0% | 1.16 [1.10, 1.22] |
| Zhu Y(woman)2024 | 1003 | 11820 | 14186 | 102979 | 0.0% | 0.58 [0.54, 0.62] |
| **Subtotal (95% CI)** | | **16445** | | **29289** | **40.7%** | **1.37 [1.16, 1.62]** |
| Total events | 3616 | | 5348 | | | |
| Heterogeneity: Tau² = 0.04; Chi² = 44.56, df = 10 (P < 0.00001); I² = 78% | | | | | | |
| Test for overall effect: Z = 3.70 (P = 0.0002) | | | | | | |
| **Total (95% CI)** | | **36799** | | **50079** | **100.0%** | **0.87 [0.72, 1.06]** |
| Total events | 10859 | | 15278 | | | |
| Heterogeneity: Tau² = 0.20; Chi² = 502.44, df = 25 (P < 0.00001); I² = 95% | | | | | | |
| Test for overall effect: Z = 1.40 (P = 0.16) | | | | | | |
| Test for subgroup differences: Chi² = 142.89, df = 3 (P < 0.00001), I² = 97.9% | | | | | | |

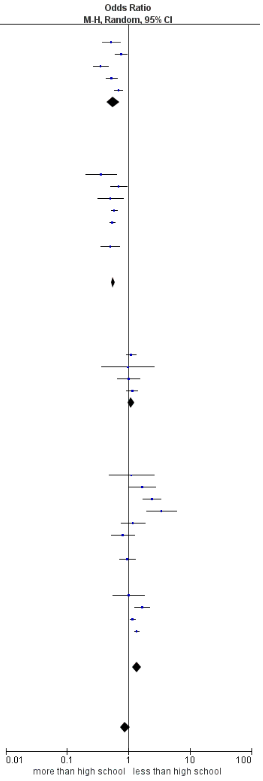


Result A

| Study or Subgroup | high school education Events | Total | less than high school Events | Total | Weight | Odds Ratio M-H, Random, 95% CI |
|---|---|---|---|---|---|---|
| **2.2.1 South Korea (SK)** | | | | | | |
| Kim HJ2022 | 144 | 602 | 175 | 632 | 4.4% | 0.82 [0.64, 1.06] |
| Lee H.J2023 | 440 | 1138 | 909 | 1933 | 5.1% | 0.71 [0.61, 0.82] |
| Seo MW(normal)2023 | 185 | 1068 | 93 | 317 | 4.1% | 0.50 [0.38, 0.67] |
| Seo MW(obese)2023 | 983 | 1487 | 462 | 619 | 4.7% | 0.66 [0.54, 0.82] |
| Yang YJ2021 | 541 | 2237 | 397 | 1409 | 5.1% | 0.81 [0.70, 0.95] |
| **Subtotal (95% CI)** | | **6532** | | **4910** | **23.4%** | **0.71 [0.61, 0.81]** |
| Total events | 2293 | | 2036 | | | |
| Heterogeneity: Tau² = 0.01; Chi² = 9.93, df = 4 (P = 0.04); I² = 60% | | | | | | |
| Test for overall effect: Z = 4.82 (P < 0.00001) | | | | | | |
| **2.2.2 the United States (US)** | | | | | | |
| Garcia D.O2022 | 70 | 102 | 50 | 90 | 2.2% | 1.75 [0.97, 3.16] |
| Hajifathalian K(less drink)2019 | 1278 | 1520 | 381 | 436 | 3.9% | 0.76 [0.56, 1.04] |
| Hajifathalian K(more drink)2019 | 508 | 635 | 128 | 150 | 2.7% | 0.69 [0.42, 1.12] |
| Jinjuvadia R2017 | 698 | 3692 | 1018 | 4370 | 5.4% | 0.77 [0.69, 0.86] |
| Liu K2024 | 750 | 2431 | 989 | 2461 | 5.3% | 0.66 [0.59, 0.75] |
| Liu Y(America)2024 | 235 | 637 | 171 | 475 | 0.0% | 1.04 [0.81, 1.33] |
| Lu Y2024 | 247 | 516 | 101 | 164 | 3.6% | 0.57 [0.40, 0.82] |
| Qi H2024 | 3372 | 6884 | 847 | 1658 | 0.0% | 0.92 [0.83, 1.02] |
| Rong F2024 | 703 | 7517 | 661 | 8476 | 0.0% | 1.22 [1.09, 1.36] |
| **Subtotal (95% CI)** | | **8896** | | **7671** | **23.1%** | **0.74 [0.63, 0.87]** |
| Total events | 3551 | | 2667 | | | |
| Heterogeneity: Tau² = 0.02; Chi² = 13.57, df = 5 (P = 0.02); I² = 63% | | | | | | |
| Test for overall effect: Z = 3.69 (P = 0.0002) | | | | | | |
| **2.2.3 other developing countries** | | | | | | |
| Alam S2018 | 139 | 472 | 545 | 1597 | 4.6% | 0.81 [0.64, 1.01] |
| Celikbilek A2018 | 14 | 26 | 47 | 98 | 1.3% | 1.27 [0.53, 3.01] |
| Mansour-Ghanaei R2019 | 228 | 545 | 123 | 265 | 4.1% | 0.83 [0.62, 1.12] |
| Sadeghianpour Z2023 | 367 | 513 | 4512 | 6249 | 4.8% | 0.97 [0.79, 1.18] |
| **Subtotal (95% CI)** | | **1556** | | **8209** | **14.8%** | **0.89 [0.78, 1.01]** |
| Total events | 748 | | 5227 | | | |
| Heterogeneity: Tau² = 0.00; Chi² = 2.28, df = 3 (P = 0.52); I² = 0% | | | | | | |
| Test for overall effect: Z = 1.80 (P = 0.07) | | | | | | |
| **2.2.4 China (CN)** | | | | | | |
| Feng RN(lean)2014 | 15 | 106 | 7 | 40 | 1.1% | 0.78 [0.29, 2.07] |
| Feng RN(obese)2014 | 145 | 200 | 46 | 73 | 2.3% | 1.55 [0.88, 2.73] |
| Li L(Cross-sectional)2020 | 111 | 711 | 60 | 375 | 3.7% | 0.97 [0.69, 1.37] |
| Li L(Longitudinal)2020 | 73 | 269 | 22 | 158 | 2.5% | 2.30 [1.36, 3.89] |
| Li Z2018 | 110 | 171 | 617 | 1060 | 3.8% | 1.29 [0.93, 1.81] |
| Liang QF2017 | 39 | 239 | 27 | 204 | 2.5% | 1.28 [0.75, 2.17] |
| Lin S2018 | 81 | 162 | 447 | 779 | 0.0% | 0.74 [0.53, 1.04] |
| Liu Y(China)2024 | 369 | 687 | 461 | 883 | 4.8% | 1.06 [0.87, 1.30] |
| Ma YB(normal)2018 | 2535 | 11684 | 3154 | 15270 | 0.0% | 1.06 [1.00, 1.13] |
| Ma YB(T2DM)2018 | 401 | 846 | 977 | 2357 | 0.0% | 1.27 [1.09, 1.49] |
| Qian X2024 | 194 | 356 | 43 | 67 | 2.4% | 0.67 [0.39, 1.15] |
| Sun T2022 | 226 | 447 | 561 | 1179 | 4.7% | 1.13 [0.91, 1.40] |
| Yu D(man)2014 | 1123 | 6838 | 889 | 5911 | 5.4% | 1.11 [1.01, 1.22] |
| Yu D(woman)2014 | 1756 | 11637 | 2615 | 19339 | 5.5% | 1.14 [1.06, 1.21] |
| Zhu Y(man)2024 | 3863 | 16934 | 6878 | 34728 | 0.0% | 1.20 [1.14, 1.25] |
| Zhu Y(woman)2024 | 3908 | 39866 | 14186 | 102979 | 0.0% | 0.68 [0.66, 0.71] |
| **Subtotal (95% CI)** | | **21661** | | **29289** | **38.7%** | **1.13 [1.04, 1.23]** |
| Total events | 4161 | | 5348 | | | |
| Heterogeneity: Tau² = 0.00; Chi² = 14.55, df = 10 (P = 0.15); I² = 31% | | | | | | |
| Test for overall effect: Z = 2.90 (P = 0.004) | | | | | | |
| **Total (95% CI)** | | **38645** | | **50079** | **100.0%** | **0.90 [0.80, 1.00]** |
| Total events | 10753 | | 15278 | | | |
| Heterogeneity: Tau² = 0.06; Chi² = 179.49, df = 25 (P < 0.00001); I² = 86% | | | | | | |
| Test for overall effect: Z = 1.92 (P = 0.05) | | | | | | |
| Test for subgroup differences: Chi² = 44.09, df = 3 (P < 0.00001), I² = 93.2% | | | | | | |

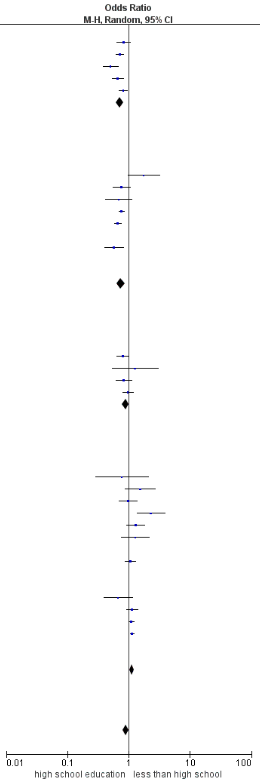


Result B

Figure S2.1: Subgroup analysis by country in Sensitive Analysis [10,23,25–49].

| Study or Subgroup | more than high school Events | more than high school Total | less than high school Events | less than high school Total | Weight | Odds Ratio M-H, Random, 95% CI |
|---|---|---|---|---|---|---|
| **5.2.1 0.0-0.4** | | | | | | |
| Feng RN(lean)2014 | 112 | 585 | 7 | 40 | 7.3% | 1.12 [0.48, 2.59] |
| Li Z2018 | 54 | 87 | 617 | 1060 | 9.2% | 1.17 [0.75, 1.84] |
| Lin S2018 | 15 | 59 | 447 | 779 | 8.5% | 0.25 [0.14, 0.46] |
| Yu D(woman)2014 | 1130 | 6456 | 2615 | 19339 | 0.0% | 1.36 [1.26, 1.46] |
| Zhu Y(woman)2024 | 1003 | 11820 | 14186 | 102979 | 10.3% | 0.58 [0.54, 0.62] |
| **Subtotal (95% CI)** | | **12551** | | **104858** | **35.2%** | **0.65 [0.38, 1.12]** |
| Total events | 1184 | | 15257 | | | |
| Heterogeneity: Tau$^2$ = 0.23; Chi$^2$ = 18.96, df = 3 (P = 0.0003); I$^2$ = 84% | | | | | | |
| Test for overall effect: Z = 1.56 (P = 0.12) | | | | | | |
| **5.2.2 0.4-0.6** | | | | | | |
| Liang QF2017 | 134 | 1227 | 27 | 204 | 9.2% | 0.80 [0.52, 1.25] |
| Liu Y(China)2024 | 112 | 220 | 461 | 883 | 9.8% | 0.95 [0.71, 1.28] |
| **Subtotal (95% CI)** | | **1447** | | **1087** | **19.0%** | **0.90 [0.71, 1.15]** |
| Total events | 246 | | 488 | | | |
| Heterogeneity: Tau$^2$ = 0.00; Chi$^2$ = 0.38, df = 1 (P = 0.54); I$^2$ = 0% | | | | | | |
| Test for overall effect: Z = 0.82 (P = 0.41) | | | | | | |
| **5.2.3 0.6-1.0** | | | | | | |
| Feng RN(obese)2014 | 573 | 775 | 46 | 73 | 9.0% | 1.66 [1.01, 2.75] |
| Li L(Cross-sectional)2020 | 159 | 508 | 60 | 375 | 9.7% | 2.39 [1.71, 3.34] |
| Li L(Longitudinal)2020 | 50 | 140 | 22 | 158 | 8.7% | 3.43 [1.95, 6.06] |
| Ma YB(normal)2018 | 3175 | 16089 | 3154 | 15270 | 0.0% | 0.94 [0.89, 1.00] |
| Ma YB(T2DM)2018 | 332 | 615 | 977 | 2357 | 0.0% | 1.66 [1.39, 1.98] |
| Qian X2024 | 115 | 179 | 43 | 67 | 8.6% | 1.00 [0.56, 1.80] |
| Sun T2022 | 153 | 254 | 561 | 1179 | 9.9% | 1.67 [1.27, 2.20] |
| Yu D(man)2014 | 1024 | 6014 | 889 | 5911 | 0.0% | 1.16 [1.05, 1.28] |
| Zhu Y(man)2024 | 2335 | 10511 | 6878 | 34728 | 0.0% | 1.16 [1.10, 1.22] |
| **Subtotal (95% CI)** | | **1856** | | **1852** | **45.7%** | **1.89 [1.36, 2.61]** |
| Total events | 1050 | | 732 | | | |
| Heterogeneity: Tau$^2$ = 0.09; Chi$^2$ = 11.69, df = 4 (P = 0.02); I$^2$ = 66% | | | | | | |
| Test for overall effect: Z = 3.82 (P = 0.0001) | | | | | | |
| **Total (95% CI)** | | **15854** | | **107797** | **100.0%** | **1.12 [0.74, 1.70]** |
| Total events | 2480 | | 16477 | | | |
| Heterogeneity: Tau$^2$ = 0.45; Chi$^2$ = 184.70, df = 10 (P < 0.00001); I$^2$ = 95% | | | | | | |
| Test for overall effect: Z = 0.53 (P = 0.60) | | | | | | |
| Test for subgroup differences: Chi$^2$ = 16.67. df = 2 (P = 0.0002). I$^2$ = 88.0% | | | | | | |

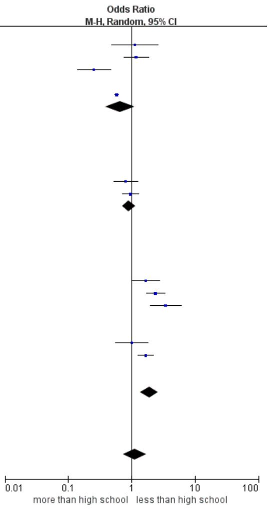


Result A

| Study or Subgroup | high school education Events | high school education Total | less than high school Events | less than high school Total | Weight | Odds Ratio M-H, Random, 95% CI |
|---|---|---|---|---|---|---|
| **6.2.1 0.0-0.4** | | | | | | |
| Feng RN(lean)2014 | 15 | 106 | 7 | 40 | 2.4% | 0.78 [0.29, 2.07] |
| Li Z2018 | 110 | 171 | 617 | 1060 | 6.7% | 1.29 [0.93, 1.81] |
| Lin S2018 | 81 | 162 | 447 | 779 | 6.6% | 0.74 [0.53, 1.04] |
| Yu D(woman)2014 | 1756 | 11637 | 2615 | 19339 | 0.0% | 1.14 [1.06, 1.21] |
| Zhu Y(woman)2024 | 3908 | 39866 | 14186 | 102979 | 8.7% | 0.68 [0.66, 0.71] |
| **Subtotal (95% CI)** | | **40305** | | **104858** | **24.4%** | **0.84 [0.60, 1.18]** |
| Total events | 4114 | | 15257 | | | |
| Heterogeneity: Tau$^2$ = 0.08; Chi$^2$ = 14.21, df = 3 (P = 0.003); I$^2$ = 79% | | | | | | |
| Test for overall effect: Z = 1.02 (P = 0.31) | | | | | | |
| **6.2.2 0.4-0.6** | | | | | | |
| Liang QF2017 | 39 | 239 | 27 | 204 | 4.9% | 1.28 [0.75, 2.17] |
| Liu Y(China)2024 | 369 | 687 | 461 | 883 | 7.9% | 1.06 [0.87, 1.30] |
| **Subtotal (95% CI)** | | **926** | | **1087** | **12.8%** | **1.09 [0.90, 1.31]** |
| Total events | 408 | | 488 | | | |
| Heterogeneity: Tau$^2$ = 0.00; Chi$^2$ = 0.41, df = 1 (P = 0.52); I$^2$ = 0% | | | | | | |
| Test for overall effect: Z = 0.87 (P = 0.38) | | | | | | |
| **6.2.3 0.6-1.0** | | | | | | |
| Feng RN(obese)2014 | 145 | 200 | 46 | 73 | 4.6% | 1.55 [0.88, 2.73] |
| Li L(Cross-sectional)2020 | 111 | 711 | 60 | 375 | 6.6% | 0.97 [0.69, 1.37] |
| Li L(Longitudinal)2020 | 73 | 269 | 22 | 158 | 5.0% | 2.30 [1.36, 3.89] |
| Ma YB(normal)2018 | 2535 | 11684 | 3154 | 15270 | 8.7% | 1.06 [1.00, 1.13] |
| Ma YB(T2DM)2018 | 401 | 846 | 977 | 2357 | 8.2% | 1.27 [1.09, 1.49] |
| Qian X2024 | 194 | 356 | 43 | 67 | 4.8% | 0.67 [0.39, 1.15] |
| Sun T2022 | 226 | 447 | 561 | 1179 | 7.7% | 1.13 [0.91, 1.40] |
| Yu D(man)2014 | 1123 | 6838 | 889 | 5911 | 8.5% | 1.11 [1.01, 1.22] |
| Zhu Y(man)2024 | 3863 | 16934 | 6878 | 34728 | 8.7% | 1.20 [1.14, 1.25] |
| **Subtotal (95% CI)** | | **38285** | | **60118** | **62.8%** | **1.15 [1.06, 1.25]** |
| Total events | 8671 | | 12630 | | | |
| Heterogeneity: Tau$^2$ = 0.01; Chi$^2$ = 24.39, df = 8 (P = 0.002); I$^2$ = 67% | | | | | | |
| Test for overall effect: Z = 3.28 (P = 0.001) | | | | | | |
| **Total (95% CI)** | | **79516** | | **166063** | **100.0%** | **1.08 [0.90, 1.29]** |
| Total events | 13193 | | 28375 | | | |
| Heterogeneity: Tau$^2$ = 0.09; Chi$^2$ = 467.15, df = 14 (P < 0.00001); I$^2$ = 97% | | | | | | |
| Test for overall effect: Z = 0.81 (P = 0.42) | | | | | | |
| Test for subgroup differences: Chi$^2$ = 3.26. df = 2 (P = 0.20). I$^2$ = 38.6% | | | | | | |

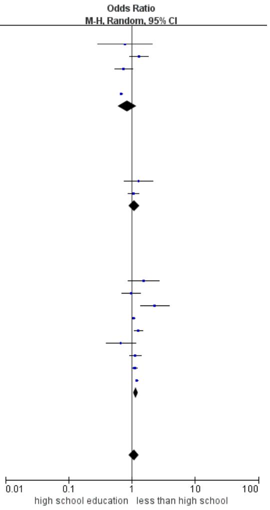


Result B

Figure S2.2: Subgroup analysis by male proportion in China in Sensitive Analysis [23,25–34].

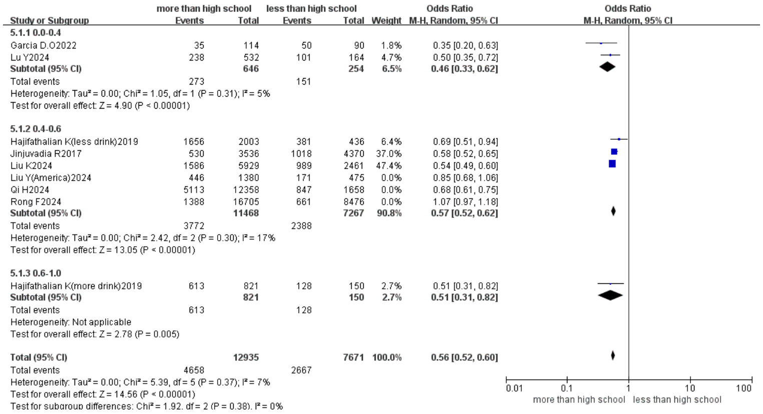

| Study or Subgroup | more than high school Events | more than high school Total | less than high school Events | less than high school Total | Weight | Odds Ratio M-H, Random, 95% CI |
|---|---|---|---|---|---|---|
| **5.1.1 0.0-0.4** | | | | | | |
| Garcia D.O2022 | 35 | 114 | 50 | 90 | 1.8% | 0.35 [0.20, 0.63] |
| Lu Y2024 | 238 | 532 | 101 | 164 | 4.7% | 0.50 [0.35, 0.72] |
| **Subtotal (95% CI)** | | **646** | | **254** | **6.5%** | **0.46 [0.33, 0.62]** |
| Total events | 273 | | 151 | | | |
| Heterogeneity: Tau² = 0.00; Chi² = 1.05, df = 1 (P = 0.31); I² = 5% | | | | | | |
| Test for overall effect: Z = 4.90 (P < 0.00001) | | | | | | |
| **5.1.2 0.4-0.6** | | | | | | |
| Hajifathalian K(less drink)2019 | 1656 | 2003 | 381 | 436 | 6.4% | 0.69 [0.51, 0.94] |
| Jinjuvadia R2017 | 530 | 3536 | 1018 | 4370 | 37.0% | 0.58 [0.52, 0.65] |
| Liu K2024 | 1586 | 5929 | 989 | 2461 | 47.4% | 0.54 [0.49, 0.60] |
| Liu Y(America)2024 | 446 | 1380 | 171 | 475 | 0.0% | 0.85 [0.68, 1.06] |
| Qi H2024 | 5113 | 12358 | 847 | 1658 | 0.0% | 0.68 [0.61, 0.75] |
| Rong F2024 | 1388 | 16705 | 661 | 8476 | 0.0% | 1.07 [0.97, 1.18] |
| **Subtotal (95% CI)** | | **11468** | | **7267** | **90.8%** | **0.57 [0.52, 0.62]** |
| Total events | 3772 | | 2388 | | | |
| Heterogeneity: Tau² = 0.00; Chi² = 2.42, df = 2 (P = 0.30); I² = 17% | | | | | | |
| Test for overall effect: Z = 13.05 (P < 0.00001) | | | | | | |
| **5.1.3 0.6-1.0** | | | | | | |
| Hajifathalian K(more drink)2019 | 613 | 821 | 128 | 150 | 2.7% | 0.51 [0.31, 0.82] |
| **Subtotal (95% CI)** | | **821** | | **150** | **2.7%** | **0.51 [0.31, 0.82]** |
| Total events | 613 | | 128 | | | |
| Heterogeneity: Not applicable | | | | | | |
| Test for overall effect: Z = 2.78 (P = 0.005) | | | | | | |
| **Total (95% CI)** | | **12935** | | **7671** | **100.0%** | **0.56 [0.52, 0.60]** |
| Total events | 4658 | | 2667 | | | |
| Heterogeneity: Tau² = 0.00; Chi² = 5.39, df = 5 (P = 0.37); I² = 7% | | | | | | |
| Test for overall effect: Z = 14.56 (P < 0.00001) | | | | | | |
| Test for subgroup differences: Chi² = 1.92, df = 2 (P = 0.38), I² = 0% | | | | | | |



Result A

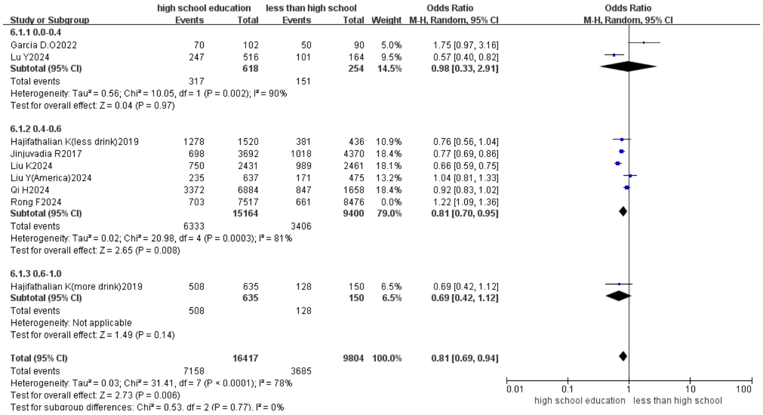

| Study or Subgroup | high school education Events | high school education Total | less than high school Events | less than high school Total | Weight | Odds Ratio M-H, Random, 95% CI |
|---|---|---|---|---|---|---|
| **6.1.1 0.0-0.4** | | | | | | |
| Garcia D.O2022 | 70 | 102 | 50 | 90 | 5.0% | 1.75 [0.97, 3.16] |
| Lu Y2024 | 247 | 516 | 101 | 164 | 9.5% | 0.57 [0.40, 0.82] |
| **Subtotal (95% CI)** | | **618** | | **254** | **14.5%** | **0.98 [0.33, 2.91]** |
| Total events | 317 | | 151 | | | |
| Heterogeneity: Tau² = 0.56; Chi² = 10.05, df = 1 (P = 0.002); I² = 90% | | | | | | |
| Test for overall effect: Z = 0.04 (P = 0.97) | | | | | | |
| **6.1.2 0.4-0.6** | | | | | | |
| Hajifathalian K(less drink)2019 | 1278 | 1520 | 381 | 436 | 10.9% | 0.76 [0.56, 1.04] |
| Jinjuvadia R2017 | 698 | 3692 | 1018 | 4370 | 18.4% | 0.77 [0.69, 0.86] |
| Liu K2024 | 750 | 2431 | 989 | 2461 | 18.1% | 0.66 [0.59, 0.75] |
| Liu Y(America)2024 | 235 | 637 | 171 | 475 | 13.2% | 1.04 [0.81, 1.33] |
| Qi H2024 | 3372 | 6884 | 847 | 1658 | 18.4% | 0.92 [0.83, 1.02] |
| Rong F2024 | 703 | 7517 | 661 | 8476 | 0.0% | 1.22 [1.09, 1.36] |
| **Subtotal (95% CI)** | | **15164** | | **9400** | **79.0%** | **0.81 [0.70, 0.95]** |
| Total events | 6333 | | 3406 | | | |
| Heterogeneity: Tau² = 0.02; Chi² = 20.98, df = 4 (P = 0.0003); I² = 81% | | | | | | |
| Test for overall effect: Z = 2.65 (P = 0.008) | | | | | | |
| **6.1.3 0.6-1.0** | | | | | | |
| Hajifathalian K(more drink)2019 | 508 | 635 | 128 | 150 | 6.5% | 0.69 [0.42, 1.12] |
| **Subtotal (95% CI)** | | **635** | | **150** | **6.5%** | **0.69 [0.42, 1.12]** |
| Total events | 508 | | 128 | | | |
| Heterogeneity: Not applicable | | | | | | |
| Test for overall effect: Z = 1.49 (P = 0.14) | | | | | | |
| **Total (95% CI)** | | **16417** | | **9804** | **100.0%** | **0.81 [0.69, 0.94]** |
| Total events | 7158 | | 3685 | | | |
| Heterogeneity: Tau² = 0.03; Chi² = 31.41, df = 7 (P < 0.0001); I² = 78% | | | | | | |
| Test for overall effect: Z = 2.73 (P = 0.006) | | | | | | |
| Test for subgroup differences: Chi² = 0.53, df = 2 (P = 0.77), I² = 0% | | | | | | |



Result B

Figure S2.3: Subgroup analysis by male proportion in the United States in Sensitive Analysis [35–42].
