# Supplementary material for: The Association Between Educational Attainment and Non-Alcoholic Fatty Liver Disease: A Systematic Review and Meta-Analysis of Observational Studies
Source: Healthcare (Basel). 2026 Apr 29;14(9):1197. doi: 10.3390/healthcare14091197 (PMC13164538; doi:10.3390/healthcare14091197)
Supplement: Supplementary file 1 [file healthcare-14-01197-s001.zip › Supplementary Material S3.pdf]

Supplementary Material S3-Results, Sensitivity Analysis, and Publication Bias

In the following content, two results are presented:

Result A is the comparison between “more-than-high-school” and “less-than-high-school”.

Result B is the comparison between “high-school-education” and “less-than-high-school”.

Part 1-Results

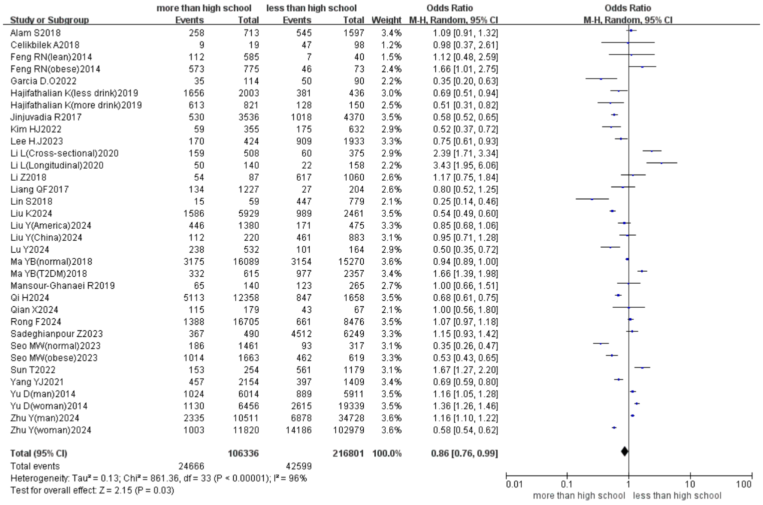

Result A

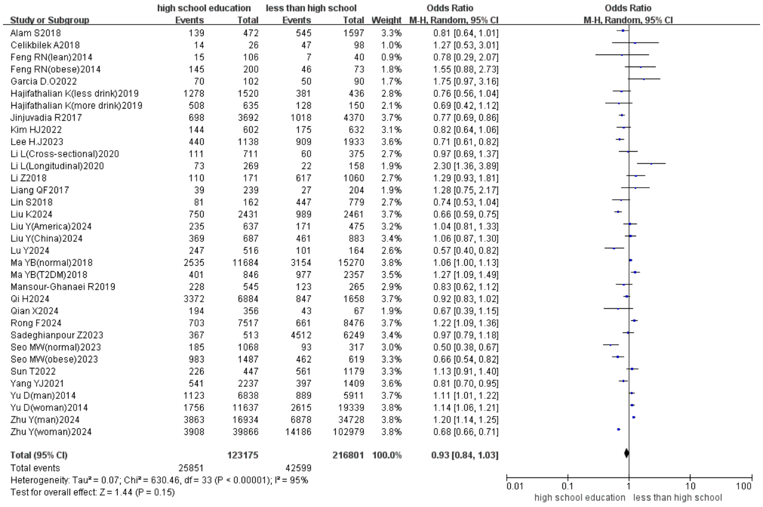

Result B

Figure 1: The association between educational attainment and NAFLD

Study or Subgroup	more than high school Events	Total	less than high school Events	Total	Weight	Odds Ratio M-H, Random, 95% CI
**2.1.1 South Korea (SK)**						
Kim HJ2022	59	355	175	632	2.9%	0.52 [0.37, 0.72]
Lee H.J2023	170	424	909	1933	3.3%	0.75 [0.61, 0.93]
Seo MW(normal)2023	186	1461	93	317	3.1%	0.35 [0.26, 0.47]
Seo MW(obese)2023	1014	1663	462	619	3.3%	0.53 [0.43, 0.65]
Yang YJ2021	457	2154	397	1409	3.4%	0.69 [0.59, 0.80]
**Subtotal (95% CI)**		**6057**		**4910**	**16.0%**	**0.56 [0.44, 0.71]**
Total events	1886		2036			
Heterogeneity: $Tau^2 = 0.06$; $Chi^2 = 22.75$, df = 4 (P = 0.0001); $I^2 = 82\%$						
Test for overall effect: Z = 4.73 (P < 0.00001)						
**2.1.2 the United States (US)**						
Garcia D.O2022	35	114	50	90	2.2%	0.35 [0.20, 0.63]
Hajifathalian K(less drink)2019	1656	2003	381	436	3.0%	0.69 [0.51, 0.94]
Hajifathalian K(more drink)2019	613	821	128	150	2.5%	0.51 [0.31, 0.82]
Jinjuvadia R2017	530	3536	1018	4370	3.5%	0.58 [0.52, 0.65]
Liu K2024	1586	5929	989	2461	3.5%	0.54 [0.49, 0.60]
Liu Y(America)2024	446	1380	171	475	3.3%	0.85 [0.68, 1.06]
Lu Y2024	238	532	101	164	2.9%	0.50 [0.35, 0.72]
Qi H2024	5113	12358	847	1658	3.5%	0.68 [0.61, 0.75]
Rong F2024	1388	16705	661	8476	3.5%	1.07 [0.97, 1.18]
**Subtotal (95% CI)**		**43378**		**18280**	**27.8%**	**0.64 [0.52, 0.79]**
Total events	11605		4346			
Heterogeneity: $Tau^2 = 0.09$; $Chi^2 = 123.52$, df = 8 (P < 0.00001); $I^2 = 94\%$						
Test for overall effect: Z = 4.07 (P < 0.0001)						
**2.1.3 other developing countries**						
Alam S2018	258	713	545	1597	3.4%	1.09 [0.91, 1.32]
Celikbilek A2018	9	19	47	98	1.2%	0.98 [0.37, 2.61]
Mansour-Ghanaei R2019	65	140	123	265	2.7%	1.00 [0.66, 1.51]
Sadeghianpour Z2023	367	490	4512	6249	3.3%	1.15 [0.93, 1.42]
**Subtotal (95% CI)**		**1362**		**8209**	**10.5%**	**1.10 [0.97, 1.26]**
Total events	699		5227			
Heterogeneity: $Tau^2 = 0.00$; $Chi^2 = 0.42$, df = 3 (P = 0.94); $I^2 = 0\%$						
Test for overall effect: Z = 1.47 (P = 0.14)						
**2.1.4 China (CN)**						
Feng RN(lean)2014	112	585	7	40	1.5%	1.12 [0.48, 2.59]
Feng RN(obese)2014	573	775	46	73	2.4%	1.66 [1.01, 2.75]
Li L(Cross-sectional)2020	159	508	60	375	2.9%	2.39 [1.71, 3.34]
Li L(Longitudinal)2020	50	140	22	158	2.2%	3.43 [1.95, 6.06]
Li Z2018	54	87	617	1060	2.6%	1.17 [0.75, 1.84]
Liang QF2017	134	1227	27	204	2.6%	0.80 [0.52, 1.25]
Lin S2018	15	59	447	779	2.1%	0.25 [0.14, 0.46]
Liu Y(China)2024	112	220	461	883	3.1%	0.95 [0.71, 1.28]
Ma YB(normal)2018	3175	16089	3154	15270	3.6%	0.94 [0.89, 1.00]
Ma YB(T2DM)2018	332	615	977	2357	3.4%	1.66 [1.39, 1.98]
Qian X2024	115	179	43	67	2.1%	1.00 [0.56, 1.80]
Sun T2022	153	254	561	1179	3.1%	1.67 [1.27, 2.20]
Yu D(man)2014	1024	6014	889	5911	3.5%	1.16 [1.05, 1.28]
Yu D(woman)2014	1130	6456	2615	19339	3.5%	1.36 [1.26, 1.46]
Zhu Y(man)2024	2335	10511	6878	34728	3.6%	1.16 [1.10, 1.22]
Zhu Y(woman)2024	1003	11820	14186	102979	3.6%	0.58 [0.54, 0.62]
**Subtotal (95% CI)**		**55539**		**185402**	**45.6%**	**1.16 [0.96, 1.40]**
Total events	10476		30990			
Heterogeneity: $Tau^2 = 0.12$; $Chi^2 = 471.64$, df = 15 (P < 0.00001); $I^2 = 97\%$						
Test for overall effect: Z = 1.53 (P = 0.13)						
**Total (95% CI)**		**106336**		**216801**	**100.0%**	**0.86 [0.76, 0.99]**
Total events	24666		42599			
Heterogeneity: $Tau^2 = 0.13$; $Chi^2 = 861.36$, df = 33 (P < 0.00001); $I^2 = 96\%$						
Test for overall effect: Z = 2.15 (P = 0.03)						
Test for subgroup differences: $Chi^2 = 40.48$, df = 3 (P < 0.00001), $I^2 = 92.6\%$						

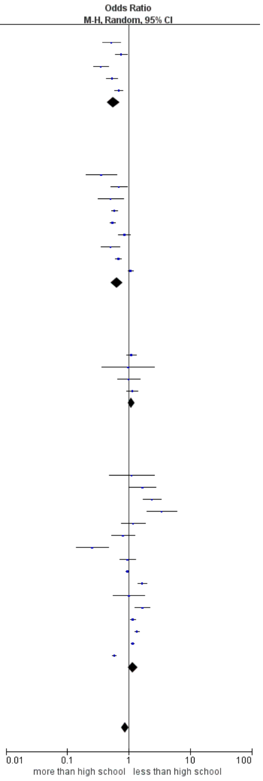

Result A

Study or Subgroup	high school education Events	Total	less than high school Events	Total	Weight	Odds Ratio M-H, Random, 95% CI
**2.2.1 South Korea (SK)**						
Kim HJ2022	144	602	175	632	3.1%	0.82 [0.64, 1.06]
Lee H.J2023	440	1138	909	1933	3.6%	0.71 [0.61, 0.82]
Seo MW(normal)2023	185	1068	93	317	3.0%	0.50 [0.38, 0.67]
Seo MW(obese)2023	983	1487	462	619	3.3%	0.66 [0.54, 0.82]
Yang YJ2021	541	2237	397	1409	3.6%	0.81 [0.70, 0.95]
**Subtotal (95% CI)**		**6532**		**4910**	**16.5%**	**0.71 [0.61, 0.81]**
Total events	2293		2036			
Heterogeneity: $Tau^2 = 0.01$; $Chi^2 = 9.93$, df = 4 (P = 0.04); $I^2 = 60\%$						
Test for overall effect: Z = 4.82 (P < 0.00001)						
**2.2.2 the United States (US)**						
Garcia D.O2022	70	102	50	90	1.7%	1.75 [0.97, 3.16]
Hajifathalian K(less drink)2019	1278	1520	381	436	2.8%	0.76 [0.56, 1.04]
Hajifathalian K(more drink)2019	508	635	128	150	2.1%	0.69 [0.42, 1.12]
Jinjuvadia R2017	698	3692	1018	4370	3.7%	0.77 [0.69, 0.86]
Liu K2024	750	2431	989	2461	3.7%	0.66 [0.59, 0.75]
Liu Y(America)2024	235	637	171	475	3.2%	1.04 [0.81, 1.33]
Lu Y2024	247	516	101	164	2.6%	0.57 [0.40, 0.82]
Qi H2024	3372	6884	847	1658	3.7%	0.92 [0.83, 1.02]
Rong F2024	703	7517	661	8476	3.7%	1.22 [1.09, 1.36]
**Subtotal (95% CI)**		**23934**		**18280**	**27.2%**	**0.86 [0.72, 1.04]**
Total events	7861		4346			
Heterogeneity: $Tau^2 = 0.06$; $Chi^2 = 76.03$, df = 8 (P < 0.00001); $I^2 = 89\%$						
Test for overall effect: Z = 1.57 (P = 0.12)						
**2.2.3 other developing countries**						
Alam S2018	139	472	545	1597	3.3%	0.81 [0.64, 1.01]
Celikbilek A2018	14	26	47	98	1.0%	1.27 [0.53, 3.01]
Mansour-Ghanaei R2019	228	545	123	265	2.9%	0.83 [0.62, 1.12]
Sadeghianpour Z2023	367	513	4512	6249	3.4%	0.97 [0.79, 1.18]
**Subtotal (95% CI)**		**1556**		**8209**	**10.6%**	**0.89 [0.78, 1.01]**
Total events	748		5227			
Heterogeneity: $Tau^2 = 0.00$; $Chi^2 = 2.28$, df = 3 (P = 0.52); $I^2 = 0\%$						
Test for overall effect: Z = 1.80 (P = 0.07)						
**2.2.4 China (CN)**						
Feng RN(lean)2014	15	106	7	40	0.9%	0.78 [0.29, 2.07]
Feng RN(obese)2014	145	200	46	73	1.8%	1.55 [0.88, 2.73]
Li L(Cross-sectional)2020	111	711	60	375	2.7%	0.97 [0.69, 1.37]
Li L(Longitudinal)2020	73	269	22	158	1.9%	2.30 [1.36, 3.89]
Li Z2018	110	171	617	1060	2.7%	1.29 [0.93, 1.81]
Liang QF2017	39	239	27	204	1.9%	1.28 [0.75, 2.17]
Lin S2018	81	162	447	779	2.7%	0.74 [0.53, 1.04]
Liu Y(China)2024	369	687	461	883	3.4%	1.06 [0.87, 1.30]
Ma YB(normal)2018	2535	11684	3154	15270	3.8%	1.06 [1.00, 1.13]
Ma YB(T2DM)2018	401	846	977	2357	3.5%	1.27 [1.09, 1.49]
Qian X2024	194	356	43	67	1.9%	0.67 [0.39, 1.15]
Sun T2022	226	447	561	1179	3.3%	1.13 [0.91, 1.40]
Yu D(man)2014	1123	6838	889	5911	3.7%	1.11 [1.01, 1.22]
Yu D(woman)2014	1756	11637	2615	19339	3.8%	1.14 [1.06, 1.21]
Zhu Y(man)2024	3863	16934	6878	34728	3.8%	1.20 [1.14, 1.25]
Zhu Y(woman)2024	3908	39866	14186	102979	3.8%	0.68 [0.66, 0.71]
**Subtotal (95% CI)**		**91153**		**185402**	**45.7%**	**1.08 [0.92, 1.27]**
Total events	14949		30990			
Heterogeneity: $Tau^2 = 0.08$; $Chi^2 = 501.22$, df = 15 (P < 0.00001); $I^2 = 97\%$						
Test for overall effect: Z = 0.93 (P = 0.35)						
**Total (95% CI)**		**123175**		**216801**	**100.0%**	**0.93 [0.84, 1.03]**
Total events	25851		42599			
Heterogeneity: $Tau^2 = 0.07$; $Chi^2 = 630.46$, df = 33 (P < 0.00001); $I^2 = 95\%$						
Test for overall effect: Z = 1.44 (P = 0.15)						
Test for subgroup differences: $Chi^2 = 15.19$, df = 3 (P = 0.002), $I^2 = 80.2\%$						

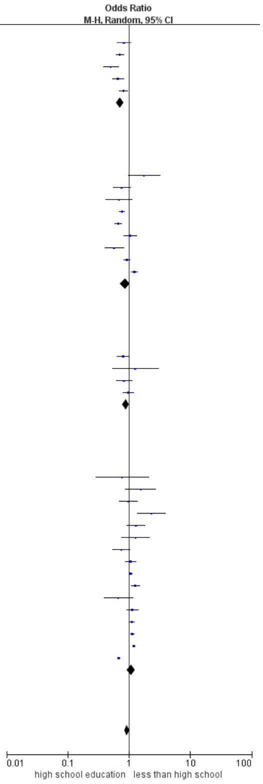

Result B

Figure 2: Subgroup analysis by country

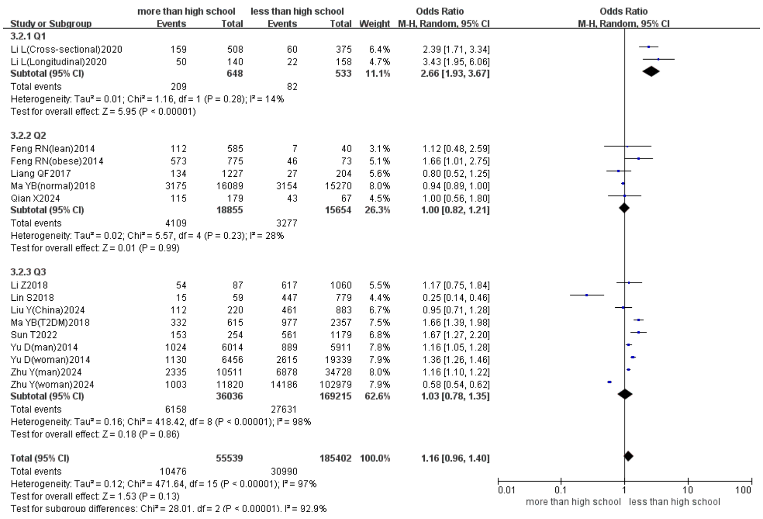

Result A

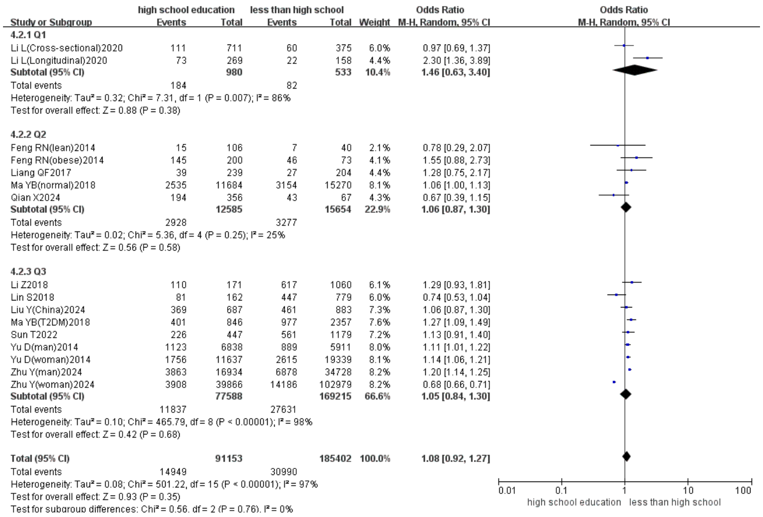

Result B

Figure 3.1: Subgroup analysis by age in China

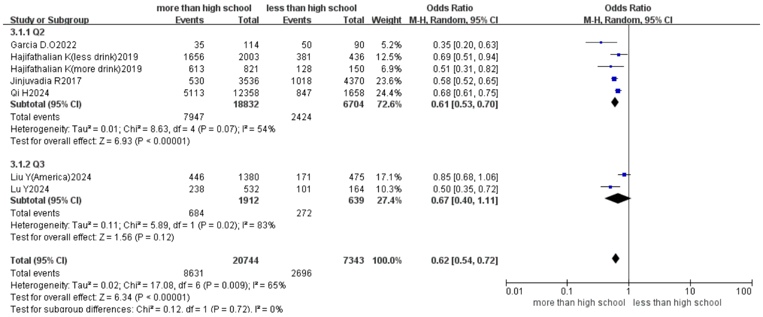

Result A

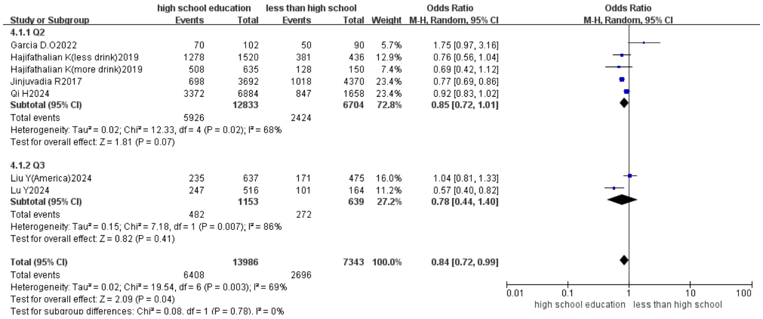

Result B

Figure 3.2: Subgroup analysis by age in the United States

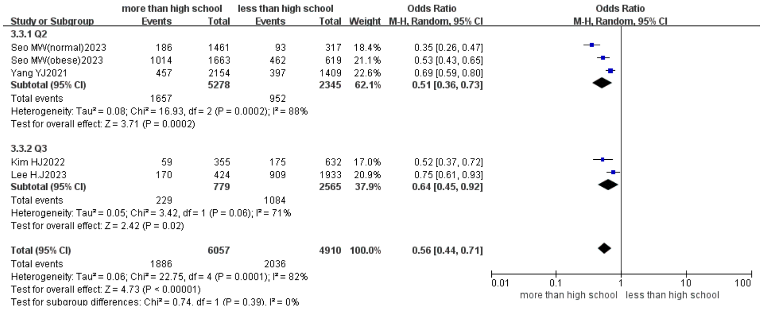

Result A

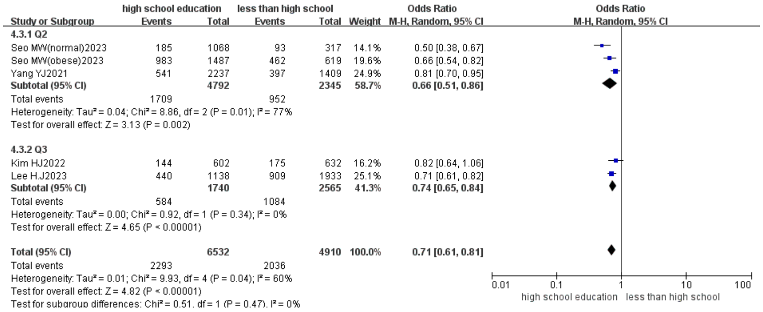

Result B

Figure 3.3: Subgroup analysis by age in South Korea

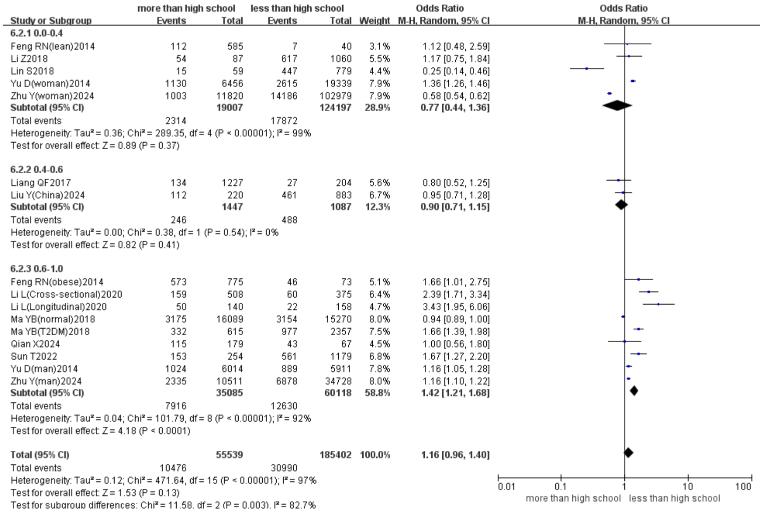

Result A

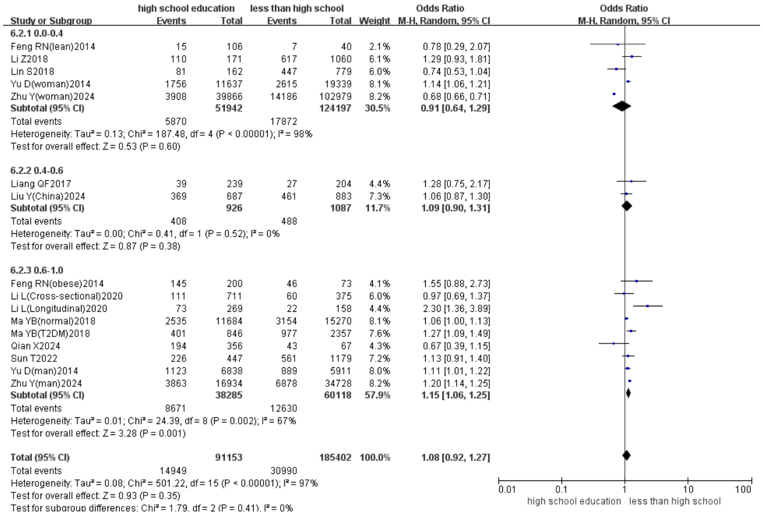

Result B

Figure 4.1: Subgroup analysis by male proportion in China

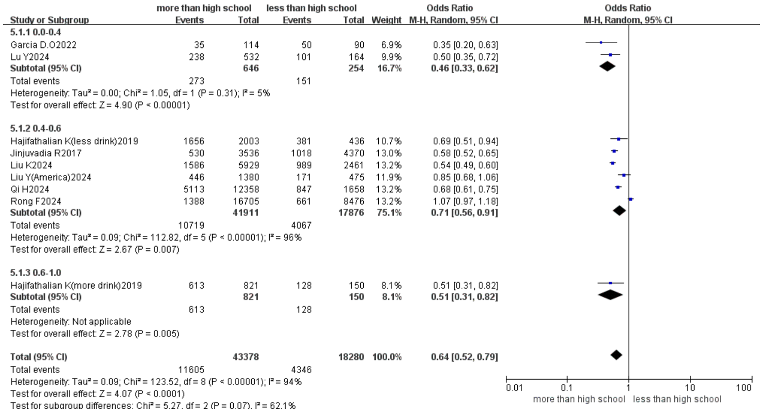

Result A

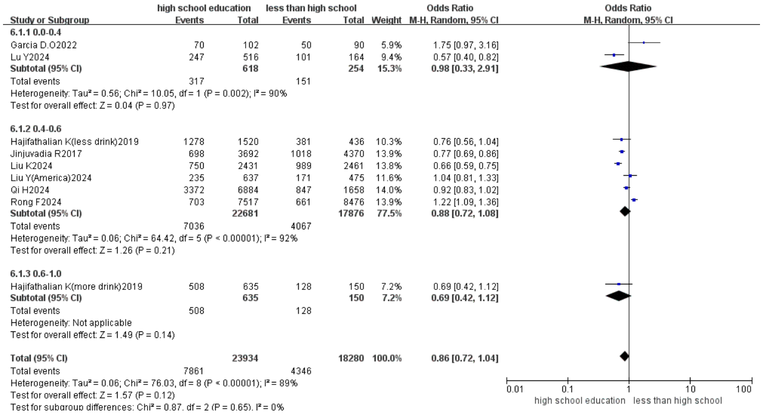

Result B

Figure 4.2: Subgroup analysis by male proportion in the United States

Part 2-Sensitive Analysis

Study or Subgroup	more than high school Events	more than high school Total	less than high school Events	less than high school Total	Weight	Odds Ratio M-H, Random, 95% CI
**2.1.1 South Korea (SK)**						
Kim HJ2022	59	355	175	632	4.0%	0.52 [0.37, 0.72]
Lee H.J2023	170	424	909	1933	4.3%	0.75 [0.61, 0.93]
Seo MW(normal)2023	186	1461	93	317	4.1%	0.35 [0.26, 0.47]
Seo MW(obese)2023	1014	1663	462	619	4.3%	0.53 [0.43, 0.65]
Yang YJ2021	457	2154	397	1409	4.4%	0.69 [0.59, 0.80]
**Subtotal (95% CI)**		**6057**		**4910**	**21.2%**	**0.56 [0.44, 0.71]**
Total events	1886		2036			
Heterogeneity: Tau² = 0.06; Chi² = 22.75, df = 4 (P = 0.0001); I² = 82%						
Test for overall effect: Z = 4.73 (P < 0.00001)						
**2.1.2 the United States (US)**						
Garcia D.O2022	35	114	50	90	3.2%	0.35 [0.20, 0.63]
Hajifathalian K(less drink)2019	1656	2003	381	436	4.1%	0.69 [0.51, 0.94]
Hajifathalian K(more drink)2019	613	821	128	150	3.5%	0.51 [0.31, 0.82]
Jinjuvadia R2017	530	3536	1018	4370	4.5%	0.58 [0.52, 0.65]
Liu K2024	1586	5929	989	2461	4.5%	0.54 [0.49, 0.60]
Liu Y(America)2024	446	1380	171	475	0.0%	0.85 [0.68, 1.06]
Lu Y2024	238	532	101	164	3.9%	0.50 [0.35, 0.72]
Qi H2024	5113	12358	847	1658	0.0%	0.68 [0.61, 0.75]
Rong F2024	1388	16705	661	8476	0.0%	1.07 [0.97, 1.18]
**Subtotal (95% CI)**		**12935**		**7671**	**23.7%**	**0.56 [0.52, 0.60]**
Total events	4658		2667			
Heterogeneity: Tau² = 0.00; Chi² = 5.39, df = 5 (P = 0.37); I² = 7%						
Test for overall effect: Z = 14.56 (P < 0.00001)						
**2.1.3 other developing countries**						
Alam S2018	258	713	545	1597	4.4%	1.09 [0.91, 1.32]
Celikbilek A2018	9	19	47	98	2.0%	0.98 [0.37, 2.61]
Mansour-Ghanaei R2019	65	140	123	265	3.7%	1.00 [0.66, 1.51]
Sadeghianpour Z2023	367	490	4512	6249	4.3%	1.15 [0.93, 1.42]
**Subtotal (95% CI)**		**1362**		**8209**	**14.4%**	**1.10 [0.97, 1.26]**
Total events	699		5227			
Heterogeneity: Tau² = 0.00; Chi² = 0.42, df = 3 (P = 0.94); I² = 0%						
Test for overall effect: Z = 1.47 (P = 0.14)						
**2.1.4 China (CN)**						
Feng RN(lean)2014	112	585	7	40	2.4%	1.12 [0.48, 2.59]
Feng RN(obese)2014	573	775	46	73	3.4%	1.66 [1.01, 2.75]
Li L(Cross-sectional)2020	159	508	60	375	4.0%	2.39 [1.71, 3.34]
Li L(Longitudinal)2020	50	140	22	158	3.2%	3.43 [1.95, 6.06]
Li Z2018	54	87	617	1060	3.6%	1.17 [0.75, 1.84]
Liang QF2017	134	1227	27	204	3.6%	0.80 [0.52, 1.25]
Lin S2018	15	59	447	779	0.0%	0.25 [0.14, 0.46]
Liu Y(China)2024	112	220	461	883	4.1%	0.95 [0.71, 1.28]
Ma YB(normal)2018	3175	16089	3154	15270	0.0%	0.94 [0.89, 1.00]
Ma YB(T2DM)2018	332	615	977	2357	0.0%	1.66 [1.39, 1.98]
Qian X2024	115	179	43	67	3.2%	1.00 [0.56, 1.80]
Sun T2022	153	254	561	1179	4.1%	1.67 [1.27, 2.20]
Yu D(man)2014	1024	6014	889	5911	4.5%	1.16 [1.05, 1.28]
Yu D(woman)2014	1130	6456	2615	19339	4.5%	1.36 [1.26, 1.46]
Zhu Y(man)2024	2335	10511	6878	34728	0.0%	1.16 [1.10, 1.22]
Zhu Y(woman)2024	1003	11820	14186	102979	0.0%	0.58 [0.54, 0.62]
**Subtotal (95% CI)**		**16445**		**29289**	**40.7%**	**1.37 [1.16, 1.62]**
Total events	3616		5348			
Heterogeneity: Tau² = 0.04; Chi² = 44.56, df = 10 (P < 0.00001); I² = 78%						
Test for overall effect: Z = 3.70 (P = 0.0002)						
**Total (95% CI)**		**36799**		**50079**	**100.0%**	**0.87 [0.72, 1.06]**
Total events	10859		15278			
Heterogeneity: Tau² = 0.20; Chi² = 502.44, df = 25 (P < 0.00001); I² = 95%						
Test for overall effect: Z = 1.40 (P = 0.16)						
Test for subgroup differences: Chi² = 142.89, df = 3 (P < 0.00001), I² = 97.9%						

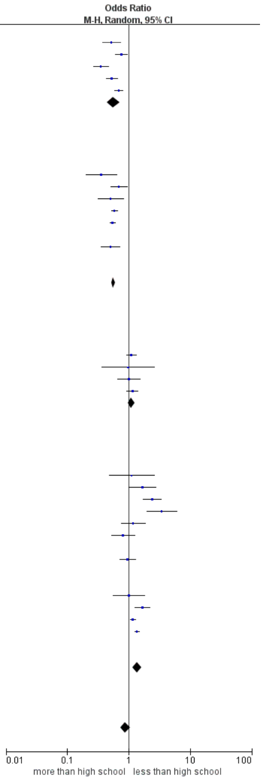

Result A

Study or Subgroup	high school education Events	high school education Total	less than high school Events	less than high school Total	Weight	Odds Ratio M-H, Random, 95% CI
**2.2.1 South Korea (SK)**						
Kim HJ2022	144	602	175	632	4.4%	0.82 [0.64, 1.06]
Lee H.J2023	440	1138	909	1933	5.1%	0.71 [0.61, 0.82]
Seo MW(normal)2023	185	1068	93	317	4.1%	0.50 [0.38, 0.67]
Seo MW(obese)2023	983	1487	462	619	4.7%	0.66 [0.54, 0.82]
Yang YJ2021	541	2237	397	1409	5.1%	0.81 [0.70, 0.95]
**Subtotal (95% CI)**		**6532**		**4910**	**23.4%**	**0.71 [0.61, 0.81]**
Total events	2293		2036			
Heterogeneity: Tau² = 0.01; Chi² = 9.93, df = 4 (P = 0.04); I² = 60%						
Test for overall effect: Z = 4.82 (P < 0.00001)						
**2.2.2 the United States (US)**						
Garcia D.O2022	70	102	50	90	2.2%	1.75 [0.97, 3.16]
Hajifathalian K(less drink)2019	1278	1520	381	436	3.9%	0.76 [0.56, 1.04]
Hajifathalian K(more drink)2019	508	635	128	150	2.7%	0.69 [0.42, 1.12]
Jinjuvadia R2017	698	3692	1018	4370	5.4%	0.77 [0.69, 0.86]
Liu K2024	750	2431	989	2461	5.3%	0.66 [0.59, 0.75]
Liu Y(America)2024	235	637	171	475	0.0%	1.04 [0.81, 1.33]
Lu Y2024	247	516	101	164	3.6%	0.57 [0.40, 0.82]
Qi H2024	3372	6884	847	1658	0.0%	0.92 [0.83, 1.02]
Rong F2024	703	7517	661	8476	0.0%	1.22 [1.09, 1.36]
**Subtotal (95% CI)**		**8896**		**7671**	**23.1%**	**0.74 [0.63, 0.87]**
Total events	3551		2667			
Heterogeneity: Tau² = 0.02; Chi² = 13.57, df = 5 (P = 0.02); I² = 63%						
Test for overall effect: Z = 3.69 (P = 0.0002)						
**2.2.3 other developing countries**						
Alam S2018	139	472	545	1597	4.6%	0.81 [0.64, 1.01]
Celikbilek A2018	14	26	47	98	1.3%	1.27 [0.53, 3.01]
Mansour-Ghanaei R2019	228	545	123	265	4.1%	0.83 [0.62, 1.12]
Sadeghianpour Z2023	367	513	4512	6249	4.8%	0.97 [0.79, 1.18]
**Subtotal (95% CI)**		**1556**		**8209**	**14.8%**	**0.89 [0.78, 1.01]**
Total events	748		5227			
Heterogeneity: Tau² = 0.00; Chi² = 2.28, df = 3 (P = 0.52); I² = 0%						
Test for overall effect: Z = 1.80 (P = 0.07)						
**2.2.4 China (CN)**						
Feng RN(lean)2014	15	106	7	40	1.1%	0.78 [0.29, 2.07]
Feng RN(obese)2014	145	200	46	73	2.3%	1.55 [0.88, 2.73]
Li L(Cross-sectional)2020	111	711	60	375	3.7%	0.97 [0.69, 1.37]
Li L(Longitudinal)2020	73	269	22	158	2.5%	2.30 [1.36, 3.89]
Li Z2018	110	171	617	1060	3.8%	1.29 [0.93, 1.81]
Liang QF2017	39	239	27	204	2.5%	1.28 [0.75, 2.17]
Lin S2018	81	162	447	779	0.0%	0.74 [0.53, 1.04]
Liu Y(China)2024	369	687	461	883	4.8%	1.06 [0.87, 1.30]
Ma YB(normal)2018	2535	11684	3154	15270	0.0%	1.06 [1.00, 1.13]
Ma YB(T2DM)2018	401	846	977	2357	0.0%	1.27 [1.09, 1.49]
Qian X2024	194	356	43	67	2.4%	0.67 [0.39, 1.15]
Sun T2022	226	447	561	1179	4.7%	1.13 [0.91, 1.40]
Yu D(man)2014	1123	6838	889	5911	5.4%	1.11 [1.01, 1.22]
Yu D(woman)2014	1756	11637	2615	19339	5.5%	1.14 [1.06, 1.21]
Zhu Y(man)2024	3863	16934	6878	34728	0.0%	1.20 [1.14, 1.25]
Zhu Y(woman)2024	3908	39866	14186	102979	0.0%	0.68 [0.66, 0.71]
**Subtotal (95% CI)**		**21661**		**29289**	**38.7%**	**1.13 [1.04, 1.23]**
Total events	4161		5348			
Heterogeneity: Tau² = 0.00; Chi² = 14.55, df = 10 (P = 0.15); I² = 31%						
Test for overall effect: Z = 2.90 (P = 0.004)						
**Total (95% CI)**		**38645**		**50079**	**100.0%**	**0.90 [0.80, 1.00]**
Total events	10753		15278			
Heterogeneity: Tau² = 0.06; Chi² = 179.49, df = 25 (P < 0.00001); I² = 86%						
Test for overall effect: Z = 1.92 (P = 0.05)						
Test for subgroup differences: Chi² = 44.09, df = 3 (P < 0.00001), I² = 93.2%						

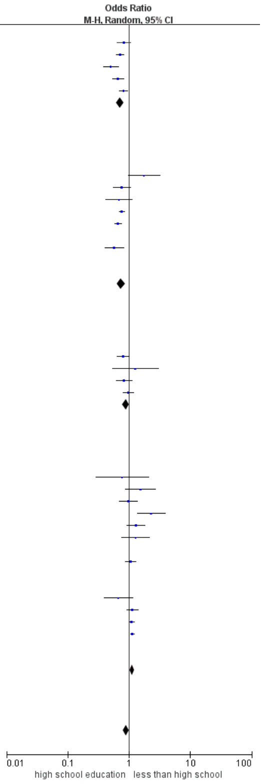

Result B

Figure 5: Subgroup analysis by country in Sensitive Analysis

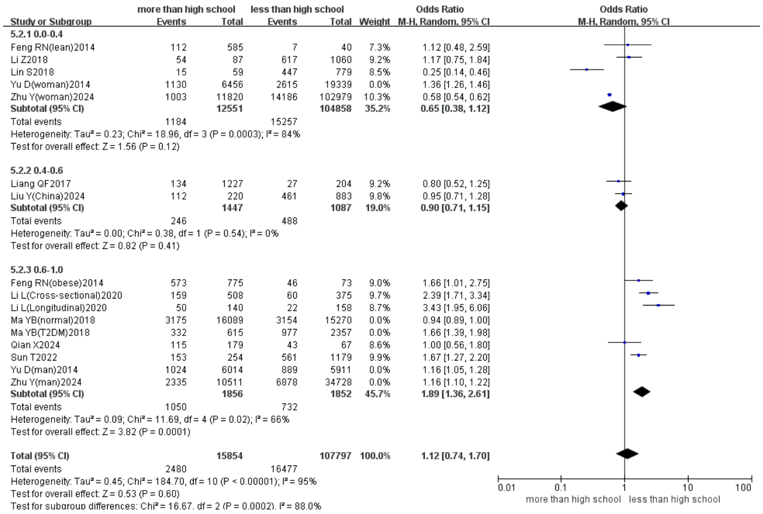

Result A

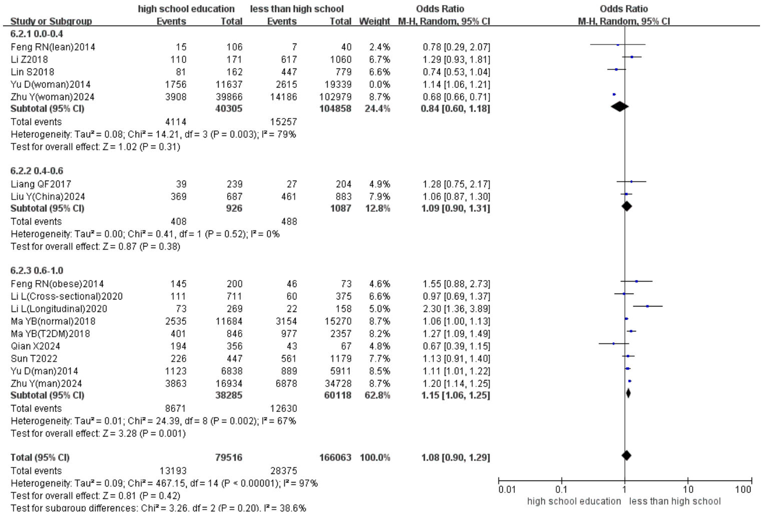

Result B

Figure 6.1: Subgroup analysis by male proportion in China in Sensitive Analysis

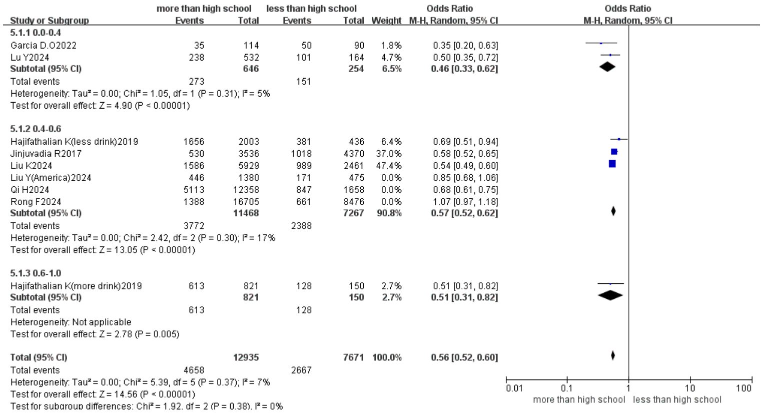

Result A

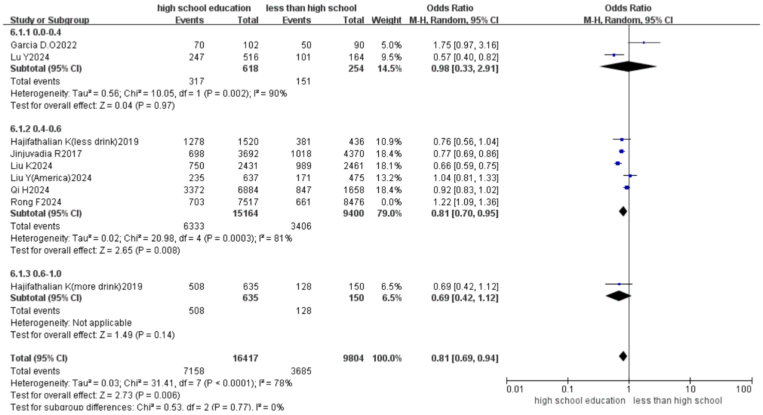

Result B

Figure 6.2: Subgroup analysis by male proportion in the United States in Sensitive Analysis

Part 3-Publication bias assessment

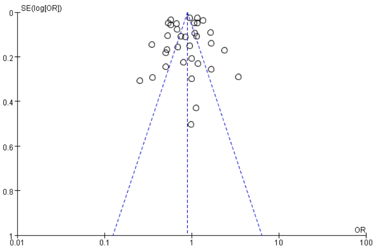

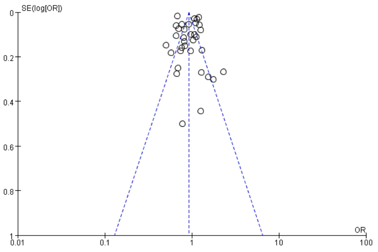

Result A Result B

Figure 7: Funnel plot of publication bias

Tests for Publication Bias

Begg's Test

```
  adj. Kendall's Score (P-Q) =      -5
          Std. Dev. of Score =   67.46
           Number of Studies =      34
                          z  =   -0.07
                   Pr > |z| =   0.941
                          z  =    0.06 (continuity corrected)
                   Pr > |z| =   0.953 (continuity corrected)
```

Egger's test

Std_Eff	Coef.	Std. Err.	t	P>|t|	[95% Conf.	Interval]
slope	-.0600548	.0949416	-0.63	0.532	-.2534446	.133335
bias	-.7867674	1.377735	-0.57	0.572	-3.593121	2.019586

Result A

Tests for Publication Bias

Begg's Test

```
  adj. Kendall's Score (P-Q) =     -15
          Std. Dev. of Score =   67.46
           Number of Studies =      34
                          z  =   -0.22
                   Pr > |z| =   0.824
                          z  =    0.21 (continuity corrected)
                   Pr > |z| =   0.836 (continuity corrected)
```

Egger's test

Std_Eff	Coef.	Std. Err.	t	P>|t|	[95% Conf.	Interval]
slope	-.0948504	.0656452	-1.44	0.158	-.2285653	.0388645
bias	.285758	1.111487	0.26	0.799	-1.978267	2.549783

Result B

Figure 8: Egger test of publication bias
